# Cluster Thinning and Vineyard Site Modulate the Metabolomic Profile of Ribolla Gialla Base and Sparkling Wines

**DOI:** 10.3390/metabo11050331

**Published:** 2021-05-20

**Authors:** Domen Škrab, Paolo Sivilotti, Piergiorgio Comuzzo, Sabrina Voce, Francesco Degano, Silvia Carlin, Panagiotis Arapitsas, Domenico Masuero, Urška Vrhovšek

**Affiliations:** 1 Department of Food Quality and Nutrition, Edmund Mach Foundation, Research and Innovation Centre, Via Edmund Mach 1, 38010 San Michele all’Adige, TN, Italy; domen.skrab@gmail.com (D.Š.); silvia.carlin@fmach.it (S.C.); panagiotis.arapitsas@fmach.it (P.A.); domenico.masuero@fmach.it (D.M.); urska.vrhovsek@fmach.it (U.V.); 2 Department of Agricultural, Food, Environmental and Animal Sciences, University of Udine, Via delle Scienze 206, 33100 Udine, UD, Italy; piergiorgio.comuzzo@uniud.it (P.C.); sabrina.voce@uniud.it (S.V.); 3 Consorzio “Friuli Colli Orientali e Ramandolo”, Piazza 27 Maggio 11, 33040 Corno di Rosazzo, UD, Italy; assistenza_tecnica@colliorientali.com

**Keywords:** Ribolla Gialla, sparkling wine, cluster thinning, vineyard site, volatile organic compounds, lipids, aromatic amino acids, sensory analysis

## Abstract

Depending on the vineyard location, cluster thinning (CT) may represent an effective tool to obtain the desired grape composition and wine quality. The effect of 20% cluster thinning on Ribolla Gialla (*Vitis vinifera* L.) sparkling wine aroma, lipid compounds, and aromatic amino acid (AAA) metabolites composition was studied for three consecutive seasons in two vineyards located in the Friuli Venezia Giulia region, Italy. In the examined sparkling wines, the vintage meteorological conditions exhibited significant influences on the metabolic profile of the samples. Data were normalized by season, and the impact of the CT treatment was evaluated for each vineyard site separately. Crop removal showed a limited positive impact on aroma compounds in sparkling wines from vineyards located in the valley. Concerning the AAA compounds, their concentration was higher in the vineyard at the foot of the hills. Cluster thinning resulted in a drop in concentration, reducing the risk of atypical aging. Despite minor differences according to targeted metabolome profiling, the sensory analysis confirmed the effects of the CT treatment in the valley floor vineyard. Reducing crop in this site, where the yield was higher, promoted a moderate improvement of Ribolla Gialla sparkling wine. In contrast, for wine produced in the vineyard at the foot of the hills, the sensory analysis indicated a preference for wines from the unthinned control samples. Overall, the study indicates that cluster thinning is a viticultural technique that could potentially improve the quality of Ribolla Gialla sparkling wines, but only in situations of excessive grape production.

## 1. Introduction

Among the indigenous white varieties cultivated in northeastern Italy, Ribolla Gialla (*Vitis vinifera* L.) is one of the most promising for producing high-quality monovarietal sparkling wines. However, due to its neutral aroma potential, some authors in previous studies focused mainly on enological practices that enhance the presence of free and glycosided forms of volatile compounds in wine throughout pre-fermentation maceration and other alternative skin contact techniques [1,2]. On the contrary, some other researchers tried to intensify the aroma profile by modifying the inoculated yeast strains [3]. To the best of our knowledge, none of the previous works have explored the impact of viticulture practices on a common metabolomic profile of Ribolla Gialla sparkling wines.

Cluster thinning is a widely used agronomic practice adopted in the vineyard with the aim to regulate the source/sink ratio, thus increasing the accumulation of the secondary metabolites [4]. In most cases, cluster thinning induces faster grape ripening, leading to higher total soluble solids, and lower titratable acidity [5]. There is a considerable body of literature dealing with crop removal as a quality tool for red grape cultivars, responding with increased anthocyanins and phenolic concentration [6], which finally promoted enhanced wine color and astringency [7,8]. Nevertheless, only a limited number of studies examined the effect of cluster removal on the aroma composition of wines [4], with contrasting results. For instance, some of these studies reported amplification of monoterpenes and esters after cluster thinning [9,10,11]. Other authors argued that the crop level had little impact on the volatile profile of wines [12,13,14]. For each grape variety, various productivity and crop loads need to be targeted to optimize grape composition. This means that such viticultural practice results positively when applied in conditions of over-crop or low crop load. At the same time, it could be ineffective or detrimental when the vines already stand in equilibrium [15]. Moreover, in the case of grapes destined for sparkling wine production, although lower sugar levels and higher titratable acidity (TA) are desirable, the excess yield could negatively affect the biosynthesis of secondary metabolites, such as aroma precursors. Thus, a yield level must be matched to obtain the best compromise between a target sugar accumulation and the biosynthesis/composition of aroma precursors in grape berries at harvest. In sparkling wine production, the yield level is normally higher to meet lower sugar accumulation, but the correct yield range needed to optimize the Ribolla Gialla sparkling wine quality still must be identified. Higher acid levels in fruit are desirable for sparkling wines since flowery and fresh aromas are preferred over greener flavors. Therefore, it can be misleading to expect lower yields in the vineyard to lead to higher wine quality, especially in warm vintages [16]. Nevertheless, a study carried out on Cavas made from *V. vinifera* cv. Parellada showed that the panel preferred wines from grapes grown in low-yielding vineyards [17]. However, despite enhancing perceived wine quality due to the increase of aroma constituents (e.g., monoterpenes) in low crop levels [18], it appears that there are conflicting data on whether the cluster thinning practice contributes positively to sparkling wine quality [16].

To our knowledge, no previous studies investigated the effect of cluster thinning on the lipid profile of sparkling wines. Lipids are important constituents in the membrane structure; they are responsible for stress adaptation and act as signaling molecules. They are also essential nutrients, and their availability can have major effects on yeast alcoholic fermentation [19]. Fatty acids represent the most important group of substances of this group of compounds, as the number of carbon atoms in their chains and the degree of saturation largely determine the effect on the wines’ organoleptic properties. Since fatty acids can also be released during winemaking fermentation, they may be present in wine in free or bound forms as ethyl esters, and they contribute with fruity characters [20]; on the contrary, a higher concentration of unsaturated fatty acids (UFAs) than saturated fatty acids (SFAs), acts as a precursor of C6 aldehydes and alcohols, causing the herbaceous flavor in the wine. Moreover, previous studies reported that medium-chain fatty acids (MCFAs) C8, C10 and C12 were negatively correlated with the foamability of sparkling wines, while the ethyl esters of C6, C8 and C10 fatty acids appeared to stabilize the foam [21]. Additionally, Pueyo et al. [22] described a positive relationship between foam stability and the total content of unsaturated linolenic acid. In contrast, saturated palmitic acid was positively related to the height of the foam collar in Cava sparkling wines.

To avoid sluggish and stuck fermentation, the course of successful fermentation kinetics in sparkling wine production can also depend on the availability of certain nitrogen compounds. Through the Ehrlich pathway, the yeasts use tryptophan (Trp), phenylalanine (Phe) and tyrosine (Tyr) to produce higher alcohols, such as tryptophol (Tol), phenyl ethanol and tyrosol (Tyl), respectively. At high concentrations, such compounds may cause forming pungent notes to both taste and smell. In contrast, at low concentrations, their contribution is more related to floral notes [23]. Through the Trp pathway, the indole metabolites can be generated. During wine aging, they participate in chemical reactions as precursors for forming other aroma substances, such as 2-aminoacetophenone (2AA) [24]. Additionally, the sulfonation of those indoles can facilitate forming off-flavors called untypical aging aroma [25]. Compared to table wines, the Trp level in sparkling wines is usually lower because two fermentations are carried out [26].

Most scientific publications in this field deal with the impact of cluster thinning on the quality of still wines, but very few have addressed how this vineyard practice affects the chemical properties of monovarietal sparkling wines. In this regard, the locally important Ribolla Gialla variety is under-researched. In addition, this study also dealt with the influence of the vineyard location on the final quality of wine from Ribolla Gialla, as in different soil conditions, yield and grape quality can be significantly affected. Therefore, this multi-targeted study was designed to monitor the changes in the composition of volatile organic compounds (VOCs), aromatic amino acid (AAA) metabolites and lipids in base and sparkling wines obtained in two different vineyards sites and with two cluster thinning treatments. Sensory analysis of the wines was performed for complementary quality assessment.

## 2. Results

### 2.1. Weather Conditions

The three seasons considered in the present investigation showed several differences in terms of temperatures and rain. However, they could be considered representative of the recent meteorological trend of the Friuli Venezia Giulia region (Supplementary Materials Figure S1).

Considering all the meteorological data together, the seasons 2017 and 2019 were similar in terms of temperatures. In terms of rainfall, the summer part of the vegetative cycles revealed similarities between the years 2018 and 2019. The particular meteorological behavior of the three seasons was responsible for some of the differences in the yield and compositional parameters described in the following paragraphs.

### 2.2. Yield and Basic Grape Quality Parameters

Yield parameters were significantly affected by all treatment, site, and season factors (Table 1). Starting with the thinning treatment, as expected, a significant reduction in the number of clusters and yield was recorded in the case of CT vines, slightly offset by a non-significant increase of the cluster weight. Moving on to the effect of the site, the average values calculated for the FG and FCO vineyards highlighted a significantly higher number of clusters and yield in the first location. The higher number of clusters registered in FG during the three seasons was counterbalanced by reducing the average cluster weight. Moreover, seasonal factors also affected the yield parameters, according to the meteorological characteristics described above. Thus, the mean number of clusters was significantly higher in the year 2018, accounting for a parallel difference in the yield parameter, partially offset by reducing the average cluster weight. On the contrary, the lack of rain that characterized the maturation period in 2019 significantly affected the cluster weight. In 2017, the same parameter was higher because of the abundance of water during the ripening period. To our knowledge, the high yield registered in the season 2018 must be considered exceptional not only for Ribolla Gialla but also for all the other varieties grown in the Friuli Venezia Giulia region [27]. This result occurred because of higher bud fertility and the meteorological course of the season that ensured optimal temperatures and rain distribution. No interaction among factors was ascertained for the yield parameters.

Alongside the production data, the technological maturation parameters of the grapes were also measured. The results revealed that cluster thinning did not significantly impact grape maturation parameters than untreated vines (UNT), even if a non-significantly higher Brix and a lower value of TA were recorded. Concerning the production site, also, in this case, Brix and TA were not significantly different. Still, the grapes of the FCO location highlighted tendentially higher values of both parameters. The lower TA analyzed in FG grapes could be explained by the different meteorological conditions of the site, even if it must be considered that the grapes were harvested 4-to-7 days earlier in the FCO location. This advance could account for the higher values of the TA analysis. The value of pH was instead significantly higher in the FG location, as it is inversely correlated with the TA [28]. A lower accumulation of total soluble solids (TSS) in the grape berries was observed in 2018 due to the higher yield of the vines. On the contrary, TA and pH were not significantly different between the seasons. Still, higher values of the former parameter were recorded in 2017 and 2018 as a result of the already mentioned higher yield that delayed the maturation dynamics.

### 2.3. Wine Basic Compositional Parameters

The alcohol content of the sparkling wines was affected by both the treatment and the season. At the same time, similar values were shown when comparing the vineyard sites (Table 2). In detail, cluster thinning had a significant, positive impact on the alcohol level, according to the accumulation of TSS in the same year. Between the seasons, the lowest values of the same parameter were recorded in 2018, with no differences between the remaining two seasons. Vineyard site and year significantly affected titratable acidity, and no effects were revealed regarding the thinning treatment. As far as the comparison between the two vineyard sites is concerned, the TA parameter was in line with grapes analysis, meaning that wines from the FG district contained a significantly lower concentration of TA compared to the wines from FCO. Additionally, the highest amount of TA was found in the wines obtained in 2017 (7.68 g/L) and 2019 (7.51 g/L), and the lowest in 2018 (7.10 g/L). Furthermore, the thinning treatment and the location factor showed no significant effect on the pH value. At the same time, seasonal differences between 2017 and 2019 were reported. Although the interaction between all factors revealed significant trends of pH between locations and treatments between the seasons, the range of variation of such parameter was limited and probably not affecting the overall quality of the wines.

### 2.4. Volatile Profile of Base Wines and Sparkling Wines

Sixty-two and sixty-six compounds, present in Ribolla Gialla base (Supplementary Materials Table S1) and sparkling wines (Supplementary Materials Table S2), respectively, were semi-quantified and separated according to their chemical classes (monoterpenes, norisoprenoids, aldehydes, alcohols, esters, acids, and ketones) and the three-ways ANOVA was applied to investigate the main effects of crop level, location of the vineyard and season.

In the case of base wines (Supplementary Materials Table S1), the CT treatment significantly affected only a very scarce number of individual aroma compounds compared to the control. Among monoterpenes, linalool was the only significantly increased compound after the thinning treatment. Similarly, the concentration of isoamyl alcohol and *cis*-3-hexenol was significantly enhanced by CT, together with the total concentration of alcohols. Concerning the other aroma classes, norisoprenoids and aldehydes were nearly unaffected by the CT treatment. At the same time, a non-significant increase in esters and reduction of acids and ketones occurred.

On the other hand, more metabolites were affected by vineyard location and mostly by the season. Several compounds revealed higher concentrations in FCO wines, including sensory-important linalool and limonene. Among norisoprenoids, β-damascenone contributed the most to the significant differences between sites and seasons. Concerning the groups of fermentation-derived compounds, an increased concentration of alcohols and aldehydes was predominantly observed in the wines originating from the FCO location. Similar findings apply to most esters, acids, and ketones, apart from methyl ethyl succinate, diethyl succinate, ethyl 9-decanoate and 2-methylthiolan-3-one significantly prevailed in the wine samples produced from the FG vineyard located in the valley floor. The statistical analysis revealed many differences related to the seasonal factor, mostly caused by the meteorological peculiarities. The concentration of most of the examined compounds was higher in the year 2017, while the lowest values were found in the season 2018 because of both the higher yield and the lower accumulation of sugars in berries.

As in the base wines, the location and mostly seasonal factor had a significant effect on the volatile profile of sparkling wines (Supplementary Materials Table S2). However, the products obtained after the secondary fermentation showed a higher degree of sensitivity to the CT treatment, with a significant increase of the concentration of citronellol, linalool and β-myrcene by 13%, 9% and 14%, respectively, than UNT. Among the aldehydes, *trans*-2-hexenal and acetaldehyde increased significantly in CT sparkling wines. A similar effect was also observed in the case of alcohols, where isoamyl alcohol, 2-phenylethanol and isobutanol accounted for the most important higher alcohols found in sparkling wines. Ethyl lactate, diethyl succinate, and ethyl-9-decanoate were shown to be the most affected esters in a positive manner. The composition of acids was similar between base wines and sparkling wines. The effect of thinning concerned mainly the concentrations of acetic acid and benzoic acid.

Significantly higher concentrations of primary aromas of monoterpenes (citronellol, linalool, and nerol) and C-13 norisoprenoids (β-damascenone and 1,1,6-trimethyl-1,2-dihydronapthalene (TDN)) were detected in the sparkling wines originating from the FCO vineyard. The FCO wines were also characterized by higher production of acetaldehyde, n-hexanol, *trans*-3-hexenol, and acetic acid. At the same time, the concentration of esters was almost unaffected by the location.

**Figure 1:**
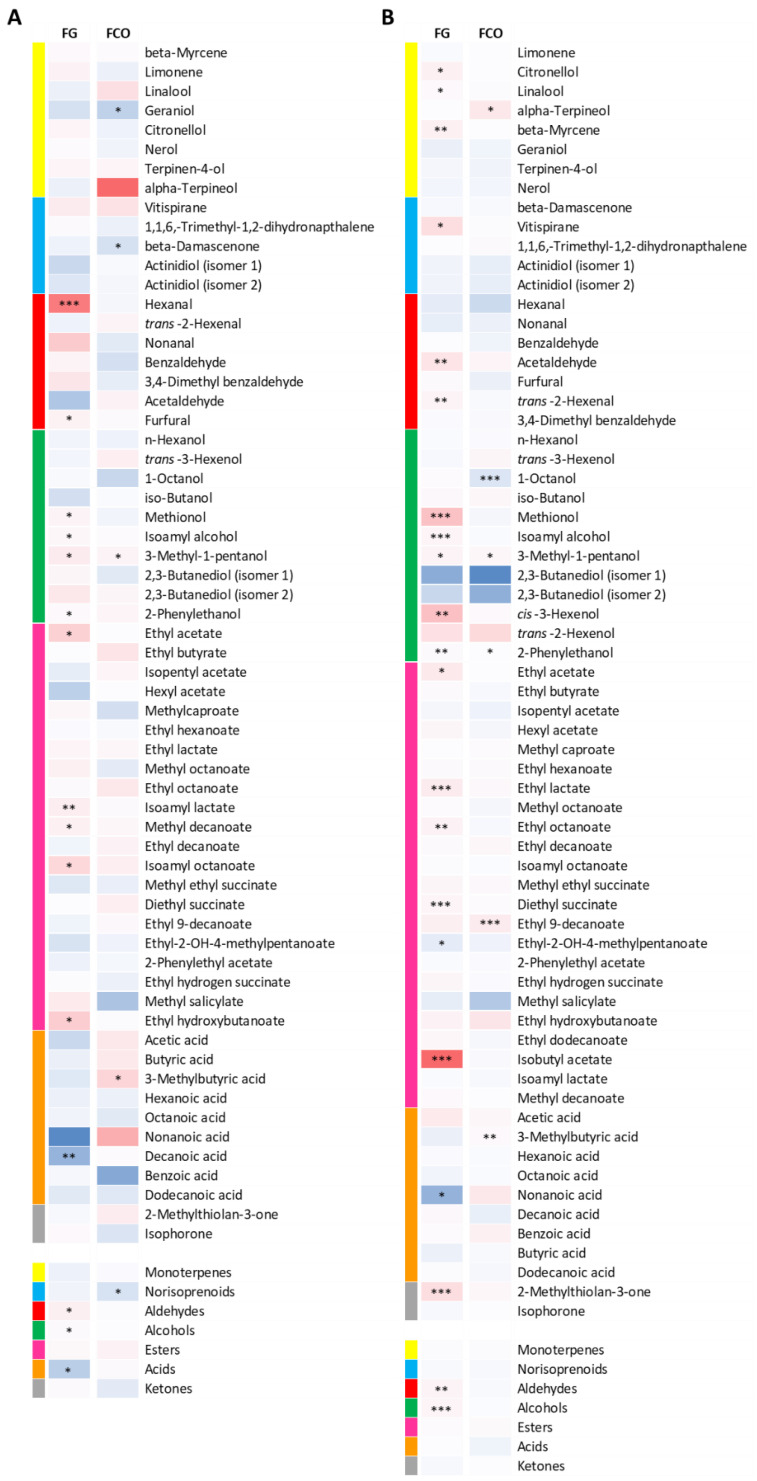
Heatmaps represent log2-fold change (CT/UNT) of the volatile compounds in the FG and FCO vineyard sites and in the base (**A**) and sparkling wines (**B**), separately. Blue and red boxes indicate lower and higher concentrations in CT, respectively. Asterisks indicate significant differences (*, *p* < 0.05; **, *p* < 0.01; ***, *p* < 0.001) between treatments after one-way ANOVA. Heatmaps were created based on the averaged values from all three vintages.

In addition to the results of the three-way ANOVA, which showed the strong effect of the vintage and only a few significant differences between treatments and locations, the main aim of the experiment was to ascertain if a yield reduction could promote an increase of aroma compounds in base and sparkling wines. To eliminate the season effect, data were normalized by season and location, and thereafter one-way ANOVA was applied (see materials and methods for details) to understand the pure effect of cluster thinning. To better show the treatment effect, data were presented in a heatmap as log2-fold change (CT/UNT) separately for FCO and FG and for the base and sparkling wines (Figure 1).

The results obtained for base wines (Figure 1A) highlighted the significantly higher effect of cluster thinning in the FG vineyard site, with increased concentration, especially for alcohols (methionol, isoamyl alcohol, 3-methyl-1-pentanol, and 2-phenylethanol), aldehydes (hexanal and furfural), and esters (ethyl acetate, isoamyl lactate, methyl decanoate, isoamyl octanoate, and ethyl hydroxybutanoate). On the contrary, a significant reduction of geraniol, β-damascenone, and total C-13 norisoprenoids was shown as an effect of thinning treatment in the case of the FCO base wines. Only 3-methyl-1-pentanol and 3-methylbutyric acid showed a significant increase in CT wines compared to the control.

Similar results were also obtained for the sparkling wines (Figure 1B), and their trend was mostly in line with what was just explained for base wines. In addition to groups of alcohols, aldehydes, and esters, citronellol, linalool, and β-myrcene from monoterpenes increased significantly in the FG samples after CT. A significantly higher concentration of vitispirane was detected in the same samples, together with *trans*-2-hexenal and *cis*-3-hexenol. Contrary to the described results, only ethyl-2-OH-4-methylpentanoate and nonanoic acid appeared to have significantly higher concentrations in UNT wines from the lowland FG vineyard site. Furthermore, the aroma composition of sparkling wines from the FCO vineyard revealed certain similarities with base wines. Even in this case, the number of statistically significant differences was noticeably smaller, with generally lower levels of substances present in the CT samples. Therefore, only α-terpineol, 3-methyl-1-pentanol, and ethyl-9-decanoate were statistically impacted by cluster thinning.

### 2.5. Lipid Profile of Base Wines and Sparkling Wines

In Supplementary Materials Tables S3 and S4, eighteen and nineteen lipid compounds were found by UHPLC/MS-MS analysis in base and sparkling wines. Most of the compounds analyzed are represented by fatty acids. At the same time, other molecules of interest belonged to the group of glycerolipids, sterols, fatty esters and prenols. Two compounds were found in higher concentration in both base and sparkling wines, in detail C16 palmitic acid and C18 stearic acid, both belonging to the group of long-chain fatty acids (LCFAs).

The analysis of variance performed on the lipids in base wines (Supplementary Materials Table S3) showed no significant impact of cluster thinning, even if small differences in concentration in favor of CT appeared in most cases. Minimal differences were also observed by comparing both wine-growing locations. It turned out that only long-chain saturated myristic acid and polyunsaturated linolenic acid were present in significantly higher concentrations in FG and FCO base wines, respectively. Even in the case of lipids, a more significant impact of the season was ascertained for fatty acids and sterols, and in general lower concentrations were reported in 2018 and 2019, while higher concentration characterized the first season of the trials.

Moving on to sparkling wines (Supplementary Materials Table S4), the analysis of variance showed that the cluster thinning treatment moderately affected the lipid concentrations, as only unsaturated palmitoleic acid appeared to be significantly different from the control (0.41 mg/L to 0.31 mg/L). However, the percentage variation between CT and UNT samples was, in general, moderate, except for lupeol, where the concentration decreased by 50% in wines after thinning. In addition, palmitoleic acid together with ergosterol was the only compound significantly different between locations. Due to low concentrations, most of the analyzed lipids did not show any trend related to the vineyard site. Furthermore, in this case, a strong impact of the seasonal factor was ascertained, with a higher concentration of lipids in the last year of the trial.

**Figure 2:**
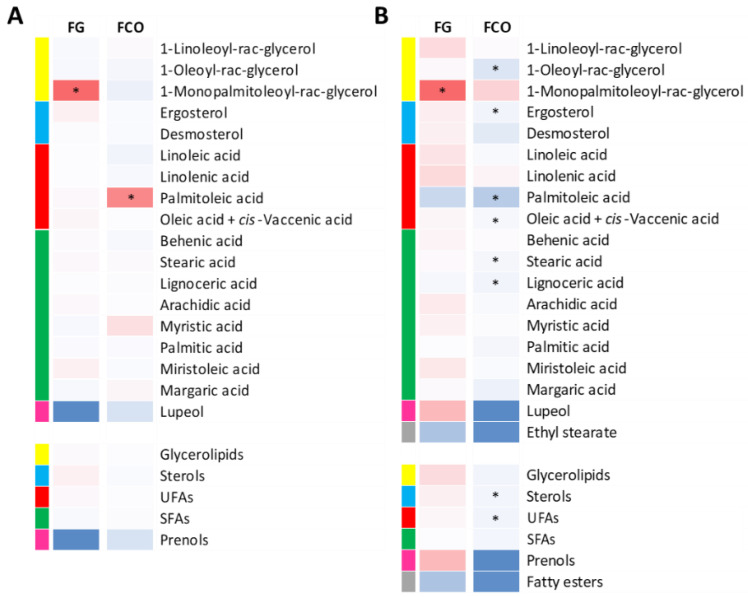
Heatmaps represent log2-fold change (CT/UNT) of the lipid compounds in the FG and FCO vineyard sites and in the base (**A**) and sparkling wines (**B**), separately. Blue and red boxes indicate lower and higher concentrations in CT, respectively. Asterisks indicate significant differences (*p* < 0.05) between treatments after one-way ANOVA. Heatmaps were created based on the averaged values from all three vintages.

As previously described for the VOCs, one-way ANOVA was performed on normalized data for the two locations separately to better understand the effect of cluster treatment. As for the base wines (Figure 2A), not much impact was ascertained since only 1-monopalmitoeoyl-rac-glycerol and palmitoleic acid resulted statistically different for FG and FCO wines, respectively. In addition, lupeol was the only prenol analyzed, and its concentration was found to be consistently higher in UNT wines in both vineyard sites, with a higher value in the FG site located on the valley floor, although the difference did not appear to be statistically significant. Conversely, two UFAs (palmitoleic acid and oleic acid + *cis*-vaccenic acid), two SFAs (stearic acid and lignoceric acid), 1-oleoyl-rac-glycerol, and ergosterol were significantly reduced by cluster thinning in the case of FCO sparkling wines, apart for 1-monopalmitoleoyl-rac-glycerol, whose concentration increased in CT wines produced in FG (Figure 2B). Moreover, ethyl stearate was additionally quantified in sparkling wines, and its concentration appeared to decrease with CT. By excluding few exceptions, the heatmap of Figure 2B more clearly shows that the effect of the CT treatment was greater in wines produced in the lowland FG area compared to the FCO wines.

### 2.6. Aromatic Amino Acid Metabolites Profile in Base Wines and Sparkling Wines

The results of AAA metabolites analysis with abscisic acid (Aba) and its glucoside (Aba-Glu) revealed a poor separation between control and CT samples in the base wines (Supplementary Materials Table S5). This being said, only N-acetyl-L-tyrosine ethyl ester (N-Tyr-EE) and indole 3-lactic acid glucoside (Ila-Glu) belonged to the class of compounds that were significantly and positively affected by the thinning treatment, as their concentrations increased in CT samples by 41% and 27%, respectively. N-acetyl serotonin (N-SER), on the other hand, decreased significantly after crop removal from 0.50 mg/L to 0.42 mg/L. In general, the amount of amino acids presents in our samples, such as Tyr and Phe, did not change by comparing UNT and CT samples, except for Trp. Furthermore, a comparison between the two locations also yielded mixed results. Tyl proved to be the most abundant compound in the FG samples, and the same was also true for Ila-Glu, phenyl lactic acid (Ph-LA), and Aba. In the FCO samples Tyr, Phe, kynurenic acid (KYNA), an indole 3-acetic acid (IAA) prevailed. The vast majority of AAA metabolites showed their statistical significance in the season factor, which leads to a greater number of interactions between year and location factors.

In sparkling wines (Supplementary Materials Table S6), the thinning treatment increased the concentration of Tyr (+40%) and N-Tyr-EE (+46%) in the CT samples, while the Trp-EE (−30%) and IAA (−39%) were significantly higher in the UNT samples. With respect to crop removal, the concentration variability was highest for those four compounds. From the results, an increase of KYNA, Tol, Ph-LA and Aba in CT was furthermore evident. At the same time, the rest of the AAA metabolites were higher in the UNT samples. Interestingly, most of the compounds prevailing in the FCO base wines maintained this ratio even after secondary fermentation, except for the phenethyl alcohols OH-Tyl and Tyr. The results of the analysis of the sparkling wine mostly coincided with the results of the base wine analysis, except for tyrosine ethyl ester (Tyr-EE), which scored 2.5 times higher concentration in FCO sparkling wines compared to the base wines, and sulfonated tryptophol (Tol-SO3H), which was additionally detected in sparkling wines. The content of Tol-SO3H was also significantly higher in the FCO wines. Considering the year factor, its high impact has been demonstrated again, as most of the analyzed compounds showed a great statistical significance. However, the only three-way interaction was identified for Tyr and Phe (*p* < 0.001 and *p* < 0.05, respectively).

**Figure 3:**
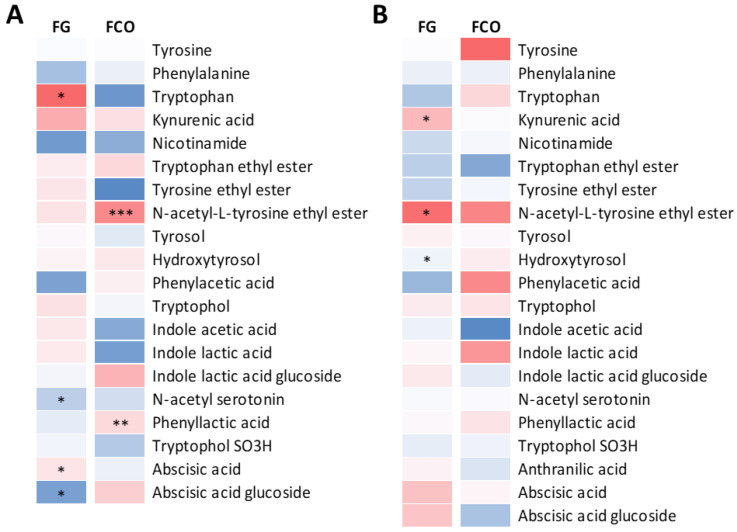
Heatmaps represent log2-fold change (CT/UNT) of the aromatic amino acid metabolites in the FG and FCO vineyard sites and in the base wines (**A**) and sparkling wines (**B**), separately. Blue and red boxes indicate lower and higher concentrations in CT, respectively. Asterisks indicate significant differences (*, *p* < 0.05; **, *p* < 0.01; ***, *p* < 0.001) between treatments after one-way ANOVA. Heatmaps were created based on the averaged values from all three vintages.

The results of the subsequent one-way ANOVA of AAA metabolites on transformed data (see Materials and Methods for details) are presented in Figure 3. By considering the vineyard sites separately, the thinning treatment in base wines (Figure 3A) resulted in a significant increase of Trp and Aba in the FG site. In contrast, the amount of N-Tyr-EE increased significantly in the FCO location. A mild increase of KYNA was shown in the FG vineyard, whether a non-significant growth of Ila-Glu was noticed in FCO. Moreover, the FG wines showed a lower concentration in UNT for N-SER and glucoside of Aba. By observing the results obtained after sparkling wines analysis (Figure 3B), only KYNA, N-Tyr-EE and phenylacetic acid (Ph-AA) resulted in statistical significance in the FG samples. However, KYNA and N-acetyl Tyr-EE only appeared to be in higher concentration in the CT wines. Meanwhile, it appeared that CT samples presented an elevated concentration of a group of compounds, including Tyr, N-Tyr-EE, Ph-AA, and Ila, as against the UNT samples.

### 2.7. Sensory Attributes

**Figure 4:**
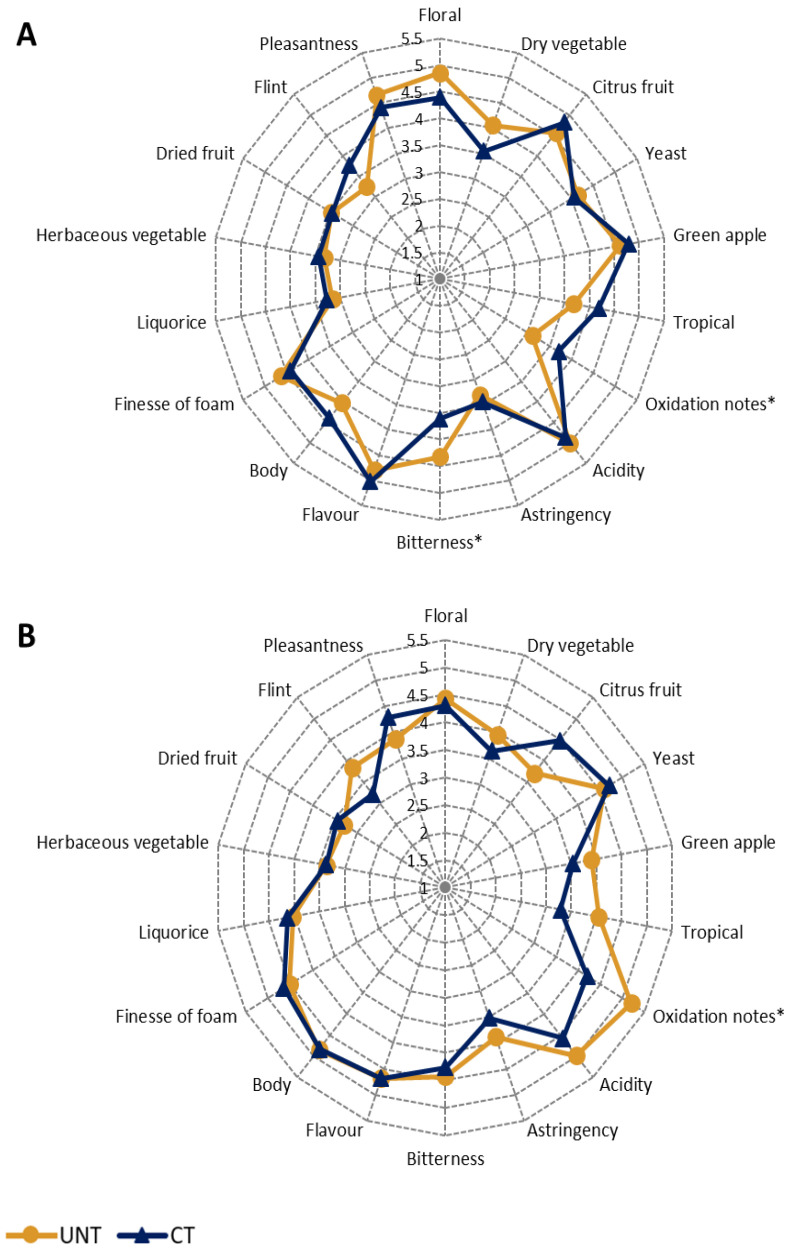
Effect of cluster thinning on the organoleptic characteristics of Ribolla Gialla sparkling wines in FCO (**A**) and FG (**B**). Average values were obtained from 2017–2019. Yellow and blue lines represent untreated (UNT) and treated (CT) samples, respectively. Asterisks (*) indicate statistical significance (*p* < 0.05) for each sensory attribute.

The sensory analysis results were normalized by the panelist before any processing and are reported in radar plots (Supplementary Materials Figures S2, S3 and Figure 4). Furthermore, in this case, a large effect of the season appeared, with the wines produced in the last season being preferred by the panel. By considering the overall effect of cluster thinning, the effects on most descriptors were negligible, with significantly lower values only for the dry vegetable character (Supplementary Materials Figure S2). On the contrary, greater differences emerged regarding different organoleptic descriptors relative to the grape production areas (Supplementary Materials Figure S3). In particular, the sparkling wines produced on the FCO site were rewarded by general pleasantness.

Furthermore, to show the effect of cluster thinning on the sensory properties of sparkling wines, data were standardized by season and site and processed through one-way ANOVA. Figure 4A shows the three-year average of the panel evaluation of the sparkling wines in the FCO area. Although not statistically significant, a higher preference for the overall pleasantness was given to the UNT wines, mostly due to significantly lower oxidation notes coupled with higher intensities of the floral and dry vegetable sensorial component. On the other hand, the mouthfeel descriptor of bitterness was significantly higher in the same UNT wines. On the contrary, CT showed a positive effect on the general pleasantness of the wines produced in the FG site (Figure 4B). Compared to the FCO location, the FG sparkling wines produced with UNT grapes were judged to be significantly more oxidized, and a higher astringency was reported. On the other hand, at the olfactory level, the CT sparkling wines showed a non-significant increase in the citrus sensation and a reduction in the tropical and green apple notes, which was combined with lower intensities of acidity perceived at mouthfeel level.

### 2.8. Multivariate Analysis of Sparkling Wines

**Figure 5:**
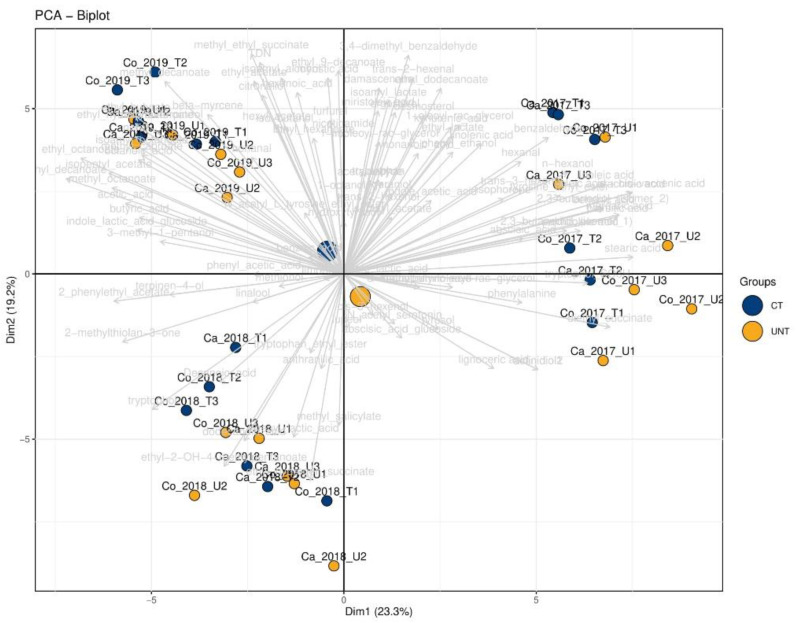
Principal component analysis biplots of sparkling wines from both vineyard sites. Small blue and yellow dots represent CT and UNT samples, respectively, and larger dots represent the centroids of respective samples. Light gray lines represent the loadings of the analyzed metabolites. Each sample label indicates the location, treatment, harvesting year and biological replicate.

The PCA biplot of all three groups of compounds detected in the sparkling wines is presented in Figure 5; looking at the spatial distribution, most of the wine samples from the 2017 harvest are located on the positive side of the PC1. Those from 2018 are present in the third quadrant. The samples from 2019 are entirely positioned in the second quadrant of the PCA biplot. After data normalization, the cluster thinning effect was observed in the FG and FCO locations independently.

**Figure 6:**
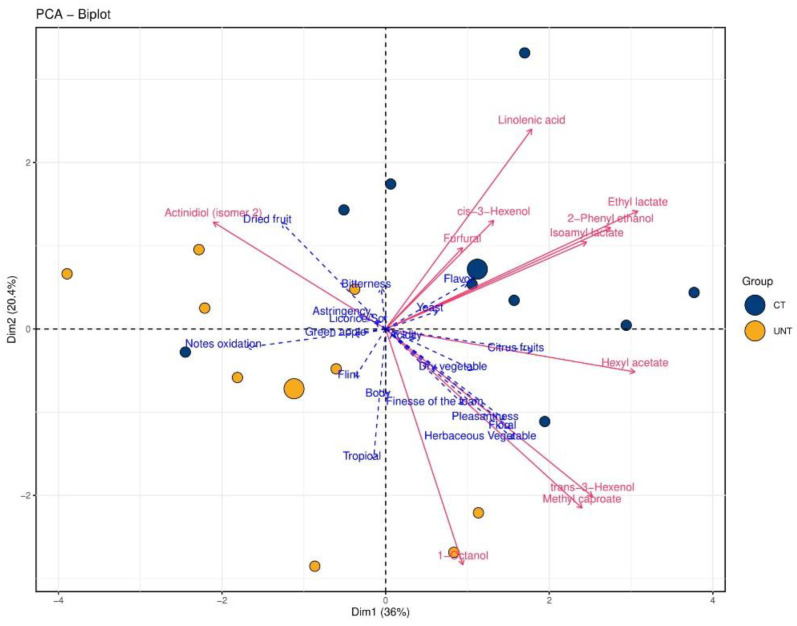
Principal component analysis biplots of sparkling wines from FG. Small blue and yellow dots represent CT and UNT samples, respectively, and larger dots represent the centroids of respective samples. Red lines represent significant loadings of volatiles, lipids, and AAA metabolites; dotted blue lines represent the loadings of sensory descriptors.

In the sparkling wines from the FG vineyard (Figure 6), the first two components accounted for 56.4% of the data explained variance, with 36% and 20.4% contribution of PC1 and PC2, respectively. Treatments were well separated, with all UNT wines mostly located on the negative side of PC1 and associated with dry fruit, tropical, oxidation notes, astringency green apple, dry vegetable, and bitterness. UNT wines were also characterized by actinidiol (isomer 2) and 1-octanol. Most CT wines were located on the positive side of PC1; they were characterized by floral, yeast, citrus fruits, body, and overall pleasantness. Pleasantness was located in the fourth quadrant, together with *trans*-3-hexenol, methyl caproate, and hexyl acetate. The rest of the ethyl esters were in the first quadrant, coupled with higher alcohols and aldehyde furfural.

**Figure 7:**
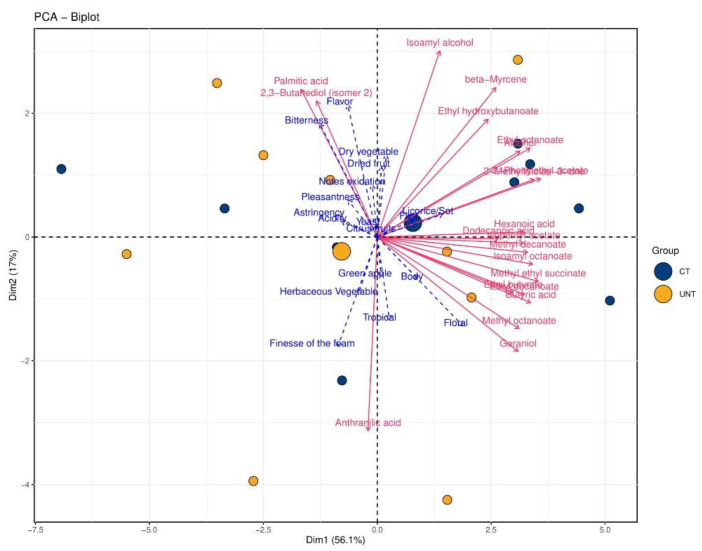
Principal component analysis biplots of sparkling wines from FCO. Small blue and yellow dots represent CT and UNT samples, respectively, and larger dots represent the centroids of respective samples. Red lines represent significant loadings of volatiles, lipids, and AAA metabolites; dotted blue lines represent the loadings of sensory descriptors.

As far as the sparkling wines from the FCO parcel are concerned, the first two PCs accounted for 73.1% of the data explained variance, with 56.1% and 17% contributions of PC1 and PC2, respectively (Figure 7). In this case, the treatments were not clearly separated. However, most of the CT wines were located on the positive side of PC1 and characterized by both negative (licorice/sotolone) and positive descriptors (citrus fruits, body, and tropical). They were correlated with most ethyl esters and monoterpenes, such as β-myrcene and geraniol. The vast majority of the UNT wines were on the negative side of PC1. The samples were associated with dried fruit and dry vegetables, as well as oxidation notes, acidity, flavor, and astringency to a less extent, and finally also with overall pleasantness. Palmitic acid and 2,3-butanediol (isomer 2) were positioned in the same quadrant as excluding pleasantness and more negative sensory descriptors. In contrast, anthranilic acid was associated with herbaceous vegetable and foam finesse.

## 3. Discussion

As already pointed out in the introduction, the effect of cluster thinning can be significantly different depending on the initial crop level. Thus, in over-crop conditions, the elimination of a certain percentage of clusters significantly improves grape quality composition, while the result could be negligible when grapevines stand in equilibrium. Moreover, the meteorological course of the seasons investigated promoted a significant impact on the biosynthesis of aroma precursors and thus on wine aromas, requiring a seasonal normalization of the data to better understand the effect of cluster thinning and location. The application of cluster thinning promoted increased VOCs concentration in the sparkling wines from the FG location. At the same time, the effect was minimal for the FCO site.

Among the monoterpenes, cluster thinning positively affected the concentration of citronellol, linalool and β-myrcene in the FG sparkling wines, in agreement with other findings [9,10,29]. Although our results show a lower concentration, it turned out that linalool and citronellol were among the most abundant terpenes in the commercial Ribolla Gialla sparkling wines [30]. Considering the effects of cluster thinning in the two locations separately, only vitispirane, among the C-13 norisoprenoids, was increased by the technique in FG sparkling wines, which contrasts with previous studies in which authors mostly found increased β-damascenone [10,31].

The CT wines from the FG location also showed higher concentrations of one of the essential sensory carbonyl compounds, acetaldehyde, which could be due to differences in Brix between locations, since acetaldehyde is considered a leakage product of the alcoholic fermentation by yeast, where sugar represents the primary substrate [32]. In this location, negligible effects of cluster thinning were ascertained on the concentration of aldehydes, in agreement with the findings of Moreno Luna et al. [14].

Concerning alcohols, cluster thinning affected 7 out of 12 compounds. The contribution of higher alcohols to wine aroma may vary from the more pleasant honey, rose, and floral attributes (e.g., 2-phenylethanol, predominantly present in the CT wines) to pungent aromas [33]. Nevertheless, the pair of higher aliphatic alcohols isobutanol and isoamyl alcohol present in the CT wines can suppress the fruity notes, but not leather, animal aromas, and therefore, play a negative role in wine aroma quality [34]. Again, cluster thinning promoted a higher concentration of alcohols when applied to the FG grapevines.

The group of esters was the most representative class of volatile compounds in this study. Two different groups of esters were dominant: the acetates of ethanol and higher alcohols and the so-called fatty acid esters that are synthesized by esterification of fatty acids with ethyl alcohol. Furthermore, for this group of compounds, cluster thinning did promote a significant increase in the FG wines, in base and mostly in sparkling wines. For instance, the concentration of isobutyl acetate was the most elevated among all the FG thinned wines. Interestingly, the same compound appeared to be an important discriminator for sparkling wines produced from the Manzoni Bianco grape variety [35]. It contributes to the sweet fruit aroma in wine [36].

The effect of cluster thinning was further researched also by analyzing the lipid composition of the samples obtained from the sparkling wines. In contrast to the base wines, the monounsaturated C16:1 palmitoleic acid appeared to be statistically impacted by both treatment and location. UFAs are required for *Saccharomyces cerevisiae* to grow under anaerobic conditions. Together with oleic acid (C18:1), palmitoleic acid represents the main UFA of *S*. *cerevisiae* [37]. The yeast autolysis, which occurred after the secondary fermentation, could, therefore, increase the amount of palmitoleic acid in the sparkling wines. While the amount of C16:1 in the sparkling wines from the FG site appeared to be higher in the control wines, the concentration of linolenic acid (C18:3) was higher in the CT wines. This compound represents one of the major components of the total lipids in grapes [38]. It is released into the grape juice, where acts as a substrate for lipoxygenase and hydroxyperoxide lyase activities, which are responsible for forming C6-aldehydes and alcohols, which contribute to the “green flavor” [39].

Fatty acids are not only important for the aroma properties of wine, but they also actively influence tactile perceptions in wines, which also includes wine foaming [40]. Different forms of fatty acid molecules can affect the stability of the foam or foam collar height in the sparkling wine in different ways. In a study on Portuguese sparkling wines [41], it was reported that the presence of surface-active monoacylglycerols of palmitic and stearic acid promoted and stabilized sparkling wine foam, while Gallart et al. [21] discovered a positive correlation between foamability and the presence of esterified fatty acids. In our case, the overall production of fatty acids and their derivatives (e.g., fatty esters) decreased in the CT Ribolla Gialla wines, regardless of location, which could consequently lead to reduced foaming or a destabilized foam collar.

Additionally, monoacylglycerols are also used in the food industry as stabilizers of foams and emulsions, so they are expected to have a certain effect on the foam properties of sparkling wines [42]. Among those, the concentration of 1-monopalmitoyl-ra-glycerol (monopalmitin) has significantly increased after the thinning treatment in FG wines. This glycerolipid is foremost present in the grape skins and seeds [43,44], but it can also be found in wine due to yeast autolysis [45].

Finally, ergosterol and desmosterol were detected among sterols. Ergosterol, in particular, is especially important, as it helps to preserve the structural integrity of yeast membranes in stressful environmental conditions, with an emphasis on yeast ethanol tolerance [46]. It may also stimulate producing esters, higher alcohols and volatile fatty acids in white wines [47]. However, our results showed a decreased number of sterols in CT sparkling wines, especially from FCO, which could lead to sluggish or stuck fermentation, which is more common during the white winemaking process, as the process of clarification can lead to heavy lipid losses [19].

In base wines, the concentration of ethyl ester of N-Tyr-EE, glucoside of Ila-Glu and of N-SER was significantly higher in the case of CT wines. N-Tyr-EE and N-SER are fermentative products of the yeast. However, their amount in the wine is determined by the number of amino acids found in grapes, such as Tyr and Trp. Moreover, N-Tyr-EE plays an active role as a Trp synthase inhibitor in regulating Trp synthesis and metabolism in yeast [48]. The Ila aglycon of hydrolyzed Ila-Glu can react with SO2 and produce the sulfonated indole Ila-SO3H. This could be of particular importance, as the sulfonation of indoles could lead to forming 2AA, which is correlated with the atypical aging off-flavor [24,25]. Different winemaking procedures, as well as wine aging, can significantly affect the amount of Ila-Glu present in wines. Arapitsas et al. [26] reported a higher concentration of Ila-Glu in young wines than sparkling wines.

A further investigation carried out in our study by employing one-way ANOVA showed some interesting results when comparing the effect of cluster thinning in two different locations. Namely, the amount of Trp in wines obtained from the FG wine-growing location was significantly higher in the CT samples than the UNT ones. This reduces the risk of atypical aging aroma formation since Trp is directly connected with the synthesis of 2AA [25]. Compared to the other white wines, Ribolla Gialla is characterized by a lower concentration of Trp compared to Malvasia [49] and Chardonnay [50], while to study Tudela et al. [51], the Trp content ranged from 6.5–574.9 µg/L in Cava sparkling wines, in line with our results.

In sparkling wines, the cluster thinning treatment led to increased the concentration of Tyr and the ethyl ester of N-acetyl-L-Tyr, while for most of the other compounds, there was a significant reduction, especially for indoleacetic acid (IAA), the ethyl ester of Trp and for Phe. The sulfonation process of IAA is largely similar to the sulfonation of Ila, described previously. As it turned out, also IAA-SO3H could promote forming aromatic aminobenzenes in wines (2AA). Such an outcome is more probable in white wines and sparkling wines compared to red wines [26]. Therefore, from the results presented in our study, it emerges that cluster thinning represents an effective tool to reduce this component by avoiding atypical aging problems. By comparing the previous results of commercial sparkling wines from the Ribolla Gialla variety [31], it has been observed that the concentration of N-acetyl-L-Tyr and IAA was higher in wines already present on the market. At the same time, the content of free Tyr and Trp-EE appeared to be greater in CT and UNT sparkling wines, respectively.

Olfactory and taste attribute rated in sparkling wines showed that significantly lower values emerged in the CT samples only for the dry vegetable sensory descriptor, considering the overall cluster thinning treatment. Furthermore, the attributes of citrus and green apple were related to the sparkling wines produced from the vineyard site close to the hills. Several authors associate the green apple descriptor with ethyl esters in wine (e.g., ethyl hexanoate), whose concentration prevailed in the FCO wines [52,53], while Alessandrini et al. [54] demonstrated that terpenes were more abundant in grapes from the high vineyard site, which agrees with our results. When the cluster thinning effect was analyzed in two separate vineyard sites, in the FCO location, overall pleasantness was assigned to the UNT samples due to their more pronounced floral aroma. Still, simultaneously, the same samples were characterized by increased bitterness of the wines, to which some esters may contribute [55]. On the contrary, general pleasantness was attributed to the CT sparkling wines from the FG vineyard site. This was most likely due to the higher amount of monoterpenes. However, the fact that oxidative notes were detected in the UNT wines also contributed to this result. According to Mayr et al. [56], the most important contributors to the oxidative off-flavor were found to be methional and 2-phenylacetaldehyde, in addition to long-chained aldehydes like *trans*-2-nonenal, *trans*-2-octenal, *trans*-2-hexenal, as well as benzaldehyde, furfural, and hexanal and some alcohols, such as 1-octen-3-ol and eugenol.

## 4. Materials and Methods

### 4.1. Chemicals and Reagents

HPLC-grade solvents dichloromethane, n-pentane, and methanol, LC–MS-grade methanol, acetonitrile, 2-propanol, chloroform, formic acid, ammonium formate, ethyl heptanoate, 1-heptanol, 2-octanol, ethyl hexanoate-d11, 3-(2-hydroxy ethyl)-indole, kynurenic acid, d-tryptophan methyl ester, L-tyrosine-ethyl ester, N-acetyl tyrosine-ethyl ester, and 3,5-di-tert-4-butylhydroxytoluene (BHT) were obtained from Sigma-Aldrich (St. Louis, MO, USA). C7–C30 n-alkane solution in n-hexane was purchased from Supelco (Bellefonte, PA, USA), while cholesterol-d7 and octadecanoic acid-d3 were obtained from CDN Isotopes (Quebec, QC, Canada). The chemical standards used to determine aromatic amino acid metabolites and lipid molecules were purchased from Aldrich-Fluka-Sigma S.r.L. (Milan, Italy), except for tryptophol-2-sulfonate (Tol-SO3H), indole-lactic acid-2-sulfonate (Ila-SO3H), and indole-acetic acid-2-sulfonate (IAA-SO3H) that were synthesized as described [26].

### 4.2. Vineyard Sites

The experiment was carried out during 2017, 2018 and 2019, in two different commercial vineyards of Ribolla Gialla, located in two different DOC districts of the FVG region. The first vineyard chosen is located in Corno di Rosazzo at the foot of the hills, sited in the Friuli Colli Orientali and Ramandolo (FCO) district (46°00′19.1″ North; 13°26′30.6″ East; elevation 94 m a.s.l.), with a planting density of 3367 plants/ha (2.7 m between the rows and 1.1 m within the row). The vineyard soil was classified as Eutric Cambisols and was characterized by a silt-clay-loam texture with no coarse. In such soil, the root penetration is limited to a depth of about 100–150 cm due to the lack of oxygen during the rainy periods of the growing season, as there is inadequate water drainage. During the summer season, the vines suffer from periods of water stress, as there is no possibility of irrigation. The second vineyard was located south of the town of Casarsa della Delizia, on the plain of Friuli Grave (FG) district (45°55′21.9″ North; 12°50′54.2″ East, 35 m a.s.l.). The planting density was 3086 plants/ha (2.7 m between rows and 1.2 m within the row). In this area, the typical soil was classified as Cutanic Luvisols. It was characterized by a silt-clay-loam texture with a low presence of skeleton. Unlike the first vineyard site, root deepening in this soil was not limited since there is adequate water drainage, even at-depth. Therefore, the conditions of anoxia are easily avoided. In addition, the water stress situations were resolved during the summer season thanks to using a drip irrigation system. In both vineyards, the clone used was the VCR 100 (Vivai Cooperativi Rauscedo, Italy) grafted onto the Kober 5BB rootstock. The training system adopted is a single arched Guyot.

### 4.3. Vine Treatments and Harvest of the Grapes

A completely randomized experimental design was set up with three replicates for each treatment compared on both vineyard sites. To optimize the choice of plants used, an evaluation of the number of clusters/vine was made in each season to correctly select the plants to use in the experiment, standardize the production where necessary, and decide the number of clusters to be removed in the cluster thinning and in the untreated plots. The treatments were applied as follows: (untreated, where the standardized production was maintained on plants (UNT), and a thinning treatment, where 20% of the production was removed from the plants at veraison stage (CT). In the vineyard in Corno di Rosazzo, CT was applied on 27 July 2017, 19 July 2018, and 02 August 2019. In the vineyard located in Casarsa della Delizia, the same treatment was performed on 4 August 2017, 23 July 2018, and 05 August 2019.

During both established experiments, the meteorological data were recorded by the ARPA–OSMER weather stations of Cividale del Friuli and San Vito al Tagliamento (ARPA FVG–OSMER, http://www.meteo.fvg.it/ (accessed on 20 May 2021)).

Grape samples were collected at harvest point from each parcel and kept in a portable fridge until arrival in the laboratory. Each sample was manually squeezed. The musts obtained were manually squeezed and used to evaluate soluble solids, titratable acidity, and pH.

### 4.4. Vinification of Base Wines

For each vineyard, approximately 30 kg of grapes × plot were harvested manually and immediately transported to the experimental winery of the University of Udine. Rotten berries were removed, and the rest of the grapes were pressed at 2 bar in an A20 pneumatic press (Grifo Marchetti, Piadena, Italy). The 15–18 L of must obtained from each vineyard plot was added to 80 mg/L of K_2_S_2_O_5_ and placed in a 50 L glass carboy, used as a fermentation vessel, to undergo the first fermentation. All the carboys were immediately inoculated with 400 mg/L of selected *Saccharomyces bayanus* commercial yeast strain Mycoferm IT07 (Ever, Pramaggiore, Italy), prepared according to the instructions of the manufacturer. The vessels were closed with airlocks to eliminate CO_2_ produced during fermentation and left at 20 °C. The fermentative activity was monitored every two days by measuring the sugar concentration of the musts. Once the concentration dropped below 10 g/L, the fermenting musts were transferred into smaller, 15 L glass carboys until alcoholic fermentation was finished. Subsequently, the base wines produced were maintained at 4 °C for about two weeks to allow the tartaric stabilization and the sedimentation of the lees.

### 4.5. Secondary Fermentation of Sparkling Wines

To produce sparkling wine with the Martinotti–Charmat method, 7 L of each base wine obtained was transferred into a stainless-steel keg. The wine was supplemented with 18 g/L of sucrose (needed to provide a 4.5 bar pressure) and 400 mg/L of the same *S*. *bayanus* yeast strain, used for the primary fermentation. Compared to the base wine, the preparation of the yeast cell suspension was slightly modified. Following rehydration in water at a temperature of 35–38 °C, an aliquot of base wine and a sucrose solution were added to better adapt the yeast to fermentative conditions. The autoclaves were sealed, saturated with carbon dioxide to avoid oxidations, and maintained at 18 °C. During fermentation, the consumption of sugars and the pressure increase were monitored continuously until the fermentation was completed after ca 40 days. Thereafter, the sparkling wine was cold stabilized at 4 °C for two weeks and bottled using an isobaric bottling machine. The sparkling wines were then stored under controlled conditions until the analysis was performed. Before the chemical analysis, all the samples were degassed for 2 min and kept at 4 °C until the extraction procedures.

### 4.6. Enological Parameters

Alcoholic strength (% *v/v*), reducing sugars (g/L), titratable acidity (g/L tartaric acid) were determined as reported in Voce et al. [31], using FTIR spectroscopy with a WineScan™ FT-120 instrument (FOSS, Hillerød, Denmark).

### 4.7. Volatile Compound Analysis

In a 20 mL headspace (HS) vial, a wine sample (1 mL) was spiked with 50 µL of 2-octanol (2.13 mg/L in ethanol) as internal standard (IS) and added to previously introduced 1.5 g of sodium chloride. Extraction of volatiles was performed by headspace solid-phase microextraction (HS–SPME) using 2 cm long 50/30 µm coated divinylbenzene/carboxen/polydimethylsiloxane (DVB/CAR/PDMS) fiber (Supelco, Sigma-Aldrich, Milan, Italy). GC–MS analysis was performed by Thermo Trace GC ultra gas chromatograph coupled to a Thermo Quantum XLS mass spectrometer (Thermo Fisher Scientific, Waltham, MA, USA), equipped with a PAL combi-xt (CTC, Zwingen, Switzerland) autosampler with an SPME option. The fiber conditioning, microextraction regime, and the configuration of the mass spectrometer were set up as previously reported by Carlin et al. [57]. Briefly, solid-phase microextraction was followed by fiber desorption in the splitless mode (3 min at 250 °C), and the fiber was reconditioned between each sample (7 min at 270 °C). The column installed in the GC oven was 30 m × 0.25 mm VF-WAXms, with a 0.25 µm film thickness (Agilent Technologies Inc., Santa Clara, CA, USA). After the sample injection, the oven temperature was initially maintained at 40 °C for 2 min and gradually ramped at 6 °C/min up to 250 °C (sustained for 5 min). The detection was carried out by electron impact mass spectrometry (MS) in total ion current (TIC) mode, using ionization energy of 70 eV and ion source temperature of 230 °C. Using ThermoXcalibur software (1.1.1.03, Thermo Scientific, Milan, Italy), the semiquantitative analysis was carried out. The final concentration of detected compounds was expressed as µg/L of the standard internal 2-octanol, considering a response factor equal to 1. Where applicable, the linear temperature-programmed retention indices were calculated (Supplementary Materials Table S7) for a series of n-alkanes (C7–C30) and compared with those reported in literature [58,59,60,61,62,63,64,65,66,67,68,69,70,71,72,73,74,75,76,77,78,79,80,81,82,83,84,85,86].

### 4.8. Lipid Compound Analysis

Lipid analysis was performed as previously described by Della Corte et al. [43], with minor modifications. A 0.5 mL aliquot of the wine sample was introduced in a 20 mL glass HS vial, with 1.5 mL of methanol, 3.0 mL of chloroform and 1.25 mL, containing BHT (500 mg/L) as an antioxidant substance, and 1.25 mL of H_2_O Milli-Q. The solution was spiked with 10 µL of stearic acid d3 (100 µg/mL) as IS. Lipids were extracted two times, and the total lower lipid-rich layer was collected and evaporated to dryness under N_2_. Afterward, the samples were reconstituted in 300 µL of acetonitrile/2-propanol/H_2_O Milli-Q (65:30:5 *v/v*/*v*), containing cholesterol d7 (1.0 µg/mL) and filtered (0.22 µm) into 2 mL HPLC amber vials with a glass insert.

UHPLC separation was performed on a Dionex 3000 chromatograph (Thermo Fisher Scientific, Waltham, MA, USA), coupled with an API 5500 triple-quadrupole mass spectrometer with an ESI source (Sciex, Concord, Vaughan, ON, Canada). Lipids were separated on a 2.7 µm, 150 × 2.1 mm RP Ascentis Express column (Sigma-Aldrich, Milan, Italy), set at 55 °C. The injection volume was 5.0 µL, and the samples were maintained in an autosampler at 10 °C during analyses. The mobile phase and chromatographic conditions are those described by Della Corte et al. [43]. Instrument control and data acquisition were performed by Analyst software (Applera Corporation, Norwalk, CT, USA), while data processing was carried out by MultiQuant, version 2.1 (Sciex, Concord, Vaughan, ON, Canada).

### 4.9. Aromatic Amino Acid Metabolites Analysis

The UHPLC-MS/MS analysis was carried out on a Waters Acquity UPLC system (Milford, MA, USA) according to Arapitsas et al. [26] with slight modifications. First, the base and sparkling wine samples were filtered at 0.22 µm by a Millex-GV filtration unit (Merc, Darmstadt, Germany) directly into 2 mL MS certified amber vials and 20 µL of the IS (10 mg 3-nitrotyrosine in 10 mL of MeOH) was added. For the separation, 1.8 µm particle size and 150 × 2.1 mm Waters Acquity HSS T3 column (Milford, MA, USA) were used. Mobile phase A consisted of H_2_O containing 0.1% formic acid, and mobile phase B was acetonitrile with 0.1% formic acid. The flow rate was 0.4 mL/min, while the column was kept at 40 °C. The injection volume was 10 µL, and the samples were stored in the autosampler at 6 °C during the analysis. Waters Xevo TQ (Milford, MA, USA) triple-quadrupole mass spectrometer with an electrospray (ESI) source was coupled to the UPLC system to perform the MS analysis. The Waters TargetLynx tools of the MassLynx 4.1 software were used for data processing. Standard solutions at eleven different concentrations were used to construct the calibration curves as adopted from Arapitsas et al. [26], except for sulfonated indole 3-lactic acid glucoside (Ila-Glu-SO3H) and Ila-Glu, which were quantified as indole 3-lactic acid (Ila).

### 4.10. Sensory Analysis

About nine months after bottling, the sparkling wines were tasted each year separately by a panel of local oenologists and producers, as well as researchers and students from the University of Udine. The median number of ten panelists was calculated, considering the three years. In the first part of the sensory evaluation, some of the wines being tasted were used to prepare the scorecard, i.e., to determine the list of sensory descriptors that characterized the sparkling wines from Ribolla Gialla. Therefore, the final list consisted of eighteen attributes: eleven referred to aroma (floral, dry vegetable, citrus fruit, yeast, green apple, tropical, oxidation notes, licorice, herbaceous vegetable, dried fruit), seven were mouthfeel attributes (acidity, astringency, bitterness, flavor, body, the finesse of foam, flint) and overall pleasantness referred to global perception. These attributes were used to develop the scorecard used by the panel for olfactory, tactile and taste characterization of the experimental wines. Samples were presented to the judges in three replicated samples each, distributing them according to a balanced, randomized service order over three subsequent sessions. Each panelist received six wines per session and was asked to evaluate a continuous 10 cm linear scale, measuring the length from the beginning of the line for each sensory attribute and sample. Sensory data were normalized twice, by panelist and by season (z-transformation) and recalculated back using the average and the standard deviation of the three-season dataset.

### 4.11. Statistical Analysis

Three-ways ANOVA was carried out using 106 JMP^®^ software (JMP 7.0, SAS Institute Inc., NC, USA) to compare the effect of cluster thinning and to assess the difference between different vineyard sites and vintages (*p* reported). When the test was significant, the averages were separated using the Student–Newman–Keuls test (*p* < 0.05). Before the data analysis, the missing values of the volatile compounds, aromatic amino acid metabolites and lipids were imputed with a random value between zero and LOQ, using a custom R script [87]. The multi-exploratory analysis was performed using principal component analysis (PCA) to evaluate the association between each group of chemical compounds and sensory variables. The R package FactoMineR v2.3 [88] was used to perform the PCA, and the packages factoextra v1.0.7 [89] and ggplot2 v 3.3.2 [90] were used to extract and visualize the result. By processing the data, a vintage effect initially emerged as dominant after the PCA analysis.

Still, to examine the impact of cluster thinning separately for the two locations, the data from all three vintages were subsequently normalized by season using the z-scale transformation, and log2 (CT/UNT) was represented in heatmaps to highlight the fold change increase/reduction of concentration in CT samples than UNT. Moreover, the significance of loadings between VOCs, lipids and aromatic amino acid metabolites was ascertained through factor analysis for each vineyard location to reduce the number of chemical parameters used in PCA. Thus, PCA analysis was repeated for each vineyard location separately, considering the just mentioned significant parameters together with sensory scores.

## 5. Conclusions

In this work, we presented the effect of cluster thinning on developing global metabolite profiles during producing monovarietal sparkling wine of the Ribolla Gialla variety. Considering the meteorological conditions during which the experimental part of this study took place, it was confirmed that the vintage had the greatest influence on the differentiation of the samples. After normalizing the results, it was possible to examine the effects of cluster thinning separately for the two production sites. The thinning of the grape clusters thus showed a minimal positive effect on the volatile composition, where the higher concentration of varietal aroma compounds (citronellol, linalool and β-myrcene) were present in the sparkling wines produced from the FG vineyard located in the valley floor. A similar outcome was achieved for the C13-norisoprenoid group of substances, where vitispirane prevailed in the FG samples. The effect of cluster thinning additionally caused the increase in metabolites associated with aromatic amino acids. In contrast to the volatile compounds, the AAA compounds, such as Trp, were predominantly present in the flat FG vineyard, which could be due to high yield and higher amount of rainfall.

At the level of organoleptic analysis, however, sparkling wines did not show any significant differences regarding overall pleasantness. At the same time, a strong effect of the production location emerged. By comparing the thinning effect in the two vineyard sites, a contrasting effect appeared in favor of the CT wines from FG and in favor of the UNT samples from the FCO site.

To sum up, cluster thinning caused slightly different effects in the two locations examined, highlighting that there may be a yield threshold above, which the wine metabolome can be significantly changed, with obvious repercussion also on the perceived organoleptic sparkling wine quality.

## Figures and Tables

**Table 1:** Yield and basic grape parameters of Ribolla Gialla grape subjected to the cluster thinning in two vineyard sites and in seasons from 2017–2019.

Parameter	Treatment (T)			Site (S)			Year (Y)				Y × T	S × T	Y × S	Y × S × T
	UNT	CT	Sig. F ^1^	FG	FCO	Sig. F	2017	2018	2019	Sig. F				
N° clusters/vine	32.89 a ^2^	25.20 b	***	35.18 a	22.91 b	***	27.13 b	31.99 a	28.02 b	**	ns	ns	ns	ns
Cluster weight (g)	190.67	200.20	ns	180.45 b	210.43 a	*	206.96 a	198.32 ab	181.03 b	***	ns	ns	ns	ns
Yield (kg/vine)	6.40 a	4.69 b	***	6.36 a	4.73 b	***	5.38 b	6.26 a	5.00 b	***	ns	ns	ns	ns
Yield (t/ha)	20.11 a	14.75 b	***	18.92 a	15.94 b	***	16.90 b	19.66 a	15.74 b	***	ns	ns	ns	ns
TSS (°Bx)	17.44	17.98	ns	17.61	17.81	ns	18.03 ab	16.79 b	18.32 a	***	ns	ns	ns	ns
TA (g/L) ^3^	6.94	6.74	ns	6.66	7.03	ns	6.61	7.11	6.81	ns	ns	ns	ns	ns
pH	3.25	3.27	ns	3.30 a	3.22 b	***	3.27	3.27	3.24	ns	ns	ns	***	ns

^1^ Data were analyzed by three-way ANOVA (ns, not significant; *, *p* < 0.05; **, *p* < 0.01; ***, *p* < 0.001), and when differences were significant, the means were separated using Tukey’s HSD test (*p* < 0.05). ^2^ Different letters (a, b) identify significantly different means. UNT—untreated control; CT—cluster thinning; FG—Friuli Grave; FCO—Friuli Colli Orientali. ^3^ TA—titratable acidity expressed in tartaric acid.

**Table 2:** Characteristics of Ribolla Gialla sparkling wine composition at different cluster thinning levels, two vineyard sites and in season from 2017–2019.

Parameter	Treatment (T)			Site (S)			Year (Y)				Y × T	S × T	Y × S	Y × S × T
	UNT	CT	Sig. F ^1^	FG	FCO	Sig. F	2017	2018	2019	Sig. F				
Alcohol (% *v*/*v*)	10.94 b ^2^	11.41 a	***	11.23	11.14	ns	11.51 a	10.42 b	11.62 a	***	ns	ns	ns	ns
Reducing sugars (g/L)	0.99 b	1.26 b	*	1.07	1.17	ns	0.20 c	1.85 a	1.32 b	***	ns	ns	ns	ns
TA (g/L) ^3^	7.50	7.36	ns	7.21 b	7.65 a	**	7.68 a	7.10 b	7.51 ab	**	ns	ns	ns	ns
pH	3.16	3.14	ns	3.16	3.14	ns	3.17 a	3.15 ab	3.12 b	*	**	ns	ns	**

^1^ Data were analyzed by three-way ANOVA (ns, not significant; *, *p* < 0.05; **, *p* < 0.01; ***, *p* < 0.001), and when differences were significant, the means were separated busing Tukey’s HSD test (*p* < 0.05). ^2^ Different letters (a, b) identify significantly different means. UNT—untreated control; CT—cluster thinning; FG—Friuli Grave; FCO—Friuli Colli Orientali. ^3^ TA—titratable acidity expressed in tartaric acid.

## Data Availability

The data presented in this study are available in the Supplementary Materials, and raw files are available on request from the corresponding author.

## Supplementary Materials

The following are available online at https://www.mdpi.com/article/10.3390/metabo11050331/s1, Figure S1: Meteorological data from 01 April to 30 September in the San Vito al Tagliamento—FG (A–C) and Cividale del Friuli—FCO (D, E, F) locations during 2017 (A, D), 2018 (B, D) and 2019 (C, F); Figure S2: Effect of cluster thinning on the organoleptic characteristics of Ribolla Gialla sparkling wines. The average values were obtained from 2017–2019 and FG-FCO vineyard sites; Figure S3: Effect of production site on the organoleptic characteristics of Ribolla Gialla sparkling wines. The average values were obtained from 2017–2019 of both UNT and CT samples; Table S1: Impact of cluster thinning treatment, vineyard site and harvest season on the volatile profile of Ribolla Gialla base wines; Table S2: Impact of cluster thinning treatment, vineyard site and harvest season on the volatile profile of Ribolla Gialla sparkling wines; Table S3: Impact of cluster thinning treatment, vineyard site and harvest season on the lipid profile of Ribolla Gialla base wines; Table S4: Impact of cluster thinning, vineyard site and harvest season on the lipid profile of Ribolla Gialla sparkling wines; Table S5: Impact of cluster thinning, vineyard site and harvest season on the aromatic amino acid metabolites profile of Ribolla Gialla base wines; Table S6: Impact of cluster thinning, vineyard site and harvest season on the aromatic amino acid metabolites profile of Ribolla Gialla sparkling wines; Table S7: Retention indices and identification method used for VOCs analysis in base wines and sparkling wines.
