# Supplementary material for: Cluster Thinning and Vineyard Site Modulate the Metabolomic Profile of Ribolla Gialla Base and Sparkling Wines
Source: Metabolites. 2021 May 20;11(5):331. doi: 10.3390/metabo11050331 (PMC8160841; doi:10.3390/metabo11050331)
Supplement: Supplementary file 1 [file metabolites-11-00331-s001.zip › metabolites-1203303-supplementary.pdf]

metabolites

Article

Cluster thinning and vineyard site modulate the metabolomic profile of Ribolla Gialla base and sparkling wines

Domen Škrab [1,2], Paolo Sivilotti [2,*], Piergiorgio Comuzzo [2], Sabrina Voce [2], Francesco Degano [3], Silvia Carlin [1], Panagiotis Arapitsas [1], Domenico Masuero [1] and Urška Vrhovšek [1]

[1] Department of Food Quality and Nutrition, Edmund Mach Foundation, Research and Innovation Centre, Via Edmund Mach 1, 38010 San Michele all'Adige, TN, Italy; domen.skrab@gmail.com (D.Š.); silvia.carlin@fmach.it (S.C.); panagiotis.arapitsas@fmach.it (P.A.); domenico.masuero@fmach.it (D.M.); urska.vrhovsek@fmach.it (U.V.)

[2] Department of Agricultural, Food, Environmental and Animal Sciences, University of Udine, Via delle Scienze 206, 33100 Udine, UD, Italy; piergiorgio.comuzzo@uniud.it (P.C.); sabrina.voce@uniud.it (S.V.)

[3] Consorzio "Friuli Colli Orientali e Ramandolo", Piazza 27 Maggio 11, 33040 Corno di Rosazzo, UD, Italy; assistenza_tecnica@colliorientali.com (F.D.)

* Correspondence: paolo.sivilotti@uniud.it (P.S.); Tel.: +39 0432 558628

Supplementary material

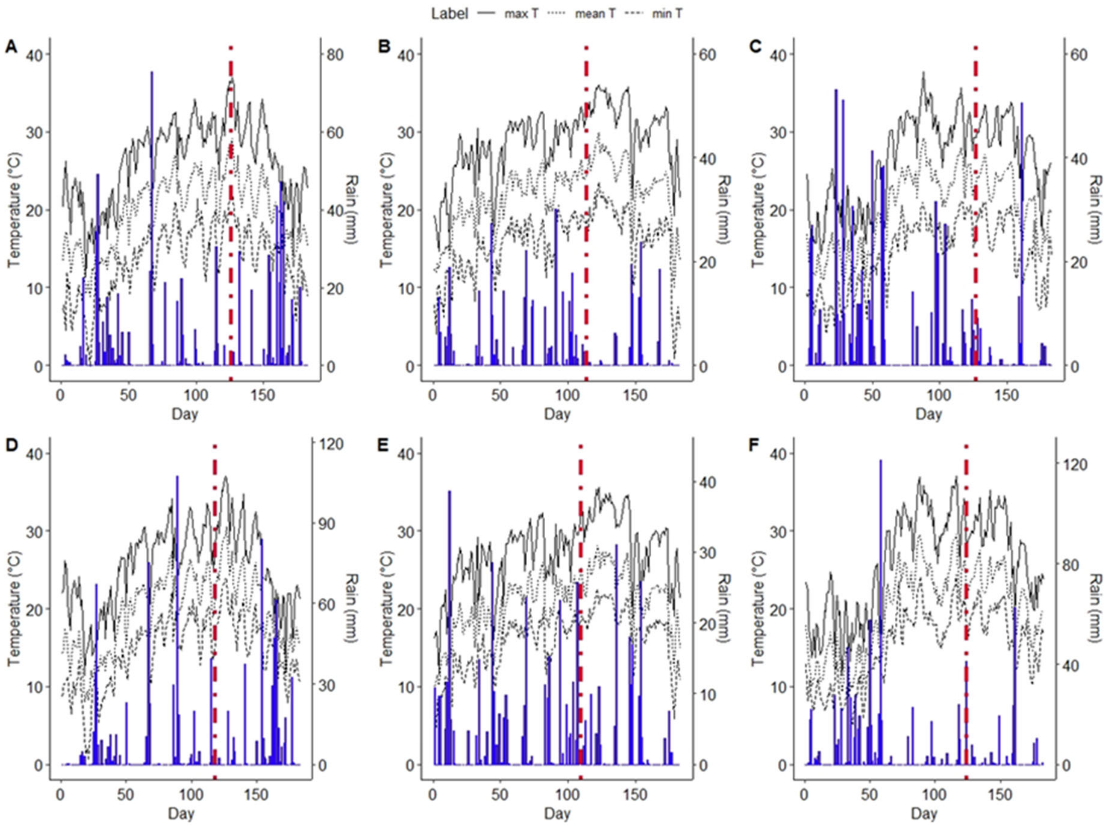

Figure S1. Meteorological data from 01 April to 30 September in the San Vito al Tagliamento—FG (**A**, **B**, **C**) and Cividale del Friuli—FCO (**D**, **E**, **F**) locations during 2017 (**A**, **D**), 2018 (**B**, **D**) and 2019 (**C**, **F**). Scattered lines represent min, mean and max temperatures while blue histograms represent rainfall. The dashed red line highlights the timing of cluster thinning in the different seasons.

Table S1. Impact of cluster thinning treatment, vineyard site and harvest season on the volatile profile of Ribolla Gialla base wines.

Compound	Treatment (T)			Site (S)			Year (Y)				Y × T	S × T	Y × S	Y × S × T
	UNT	CT	Sig. F [1]	FG	FCO	Sig. F	2017	2018	2019	Sig. F				
Monoterpenes	18.87	19.26	ns	18.83	19.30	ns	21.21a	19.44ab [2]	16.54b	***	ns	ns	*	*
β-myrcene	0.31	0.33	ns	0.29	0.35	ns	0.59a	0.20b	0.17b	***	ns	ns	ns	ns
Limonene	1.31	1.40	ns	1.24b	1.47a	**	0.57c	1.37b	2.13a	***	*	ns	ns	*
Linalool	3.76b	4.26a	*	3.81b	4.22a	*	4.58a	4.77a	2.69b	***	*	**	*	**
Geraniol	7.35	6.30	ns	6.68	6.96	ns	7.88a	5.99b	6.59ab	*	ns	ns	ns	ns
Citronellol	3.70	3.81	ns	3.66	3.85	ns	4.75a	3.54b	2.98b	***	ns	ns	***	ns
Nerol	1.51	1.57	ns	1.52	1.56	ns	1.53	1.65	1.43	ns	**	*ns*	**	**
Terpinen-4-ol	0.24	0.28	ns	0.25	0.27	ns	0.30a	0.30a	0.19b	**	ns	ns	ns	ns
α-Terpineol	0.79	1.31	ns	1.38	0.72	ns	1.01	1.62	0.51	ns	ns	ns	ns	ns
Norisoprenoids	13.29	12.88	ns	11.20b	14.97a	***	19.68a	11.76b	7.82c	**	ns	ns	***	ns
Vitispirane	0.37	0.47	ns	0.44	0.40	ns	0.70a	0.22b	0.34b	***	ns	ns	ns	ns
TDN	0.67	0.69	ns	0.66	0.70	ns	0.64	0.75	0.65	ns	ns	ns	ns	ns
β-Damascenone	11.95	11.46	ns	9.87b	13.54a	***	17.88a	10.53b	6.71c	***	ns	ns	***	ns
Actinidiol (isomer 1)	0.17	0.14	ns	0.12	0.20	ns	0.23a	0.18a	0.06b	*	ns	ns	ns	ns
Actinidiol (isomer 2)	0.12	0.12	ns	0.11b	0.14a	**	0.23a	0.08b	0.06c	***	ns	ns	*	ns
Aldehydes	116.94	131.80	ns	113.25b	135.49a	*	181.26a	98.29b	93.56b	***	ns	ns	**	ns
Hexanal	0.43	0.69	ns	0.45	0.68	ns	1.02a	0.34b	0.33b	***	ns	ns	**	ns
trans-2-Hexenal	84.88	89.28	ns	91.43	82.73	ns	108.56a	71.83b	80.85ab	*	ns	ns	ns	ns
Nonanal	14.63	26.22	ns	6.41b	34.44a	**	45.71a	10.51b	5.05b	***	ns	ns	**	ns
Benzaldehyde	6.16	5.51	ns	4.66	7.00	ns	10.99a	4.26b	2.25b	**	ns	ns	ns	ns
3,4-Dimethyl benzaldehyde	0.69	0.60	ns	0.36b	0.93a	*	0.49b	1.17a	0.28b	*	ns	ns	*	ns
Acetaldehyde	9.71	9.01	ns	9.51	9.20	ns	13.42a	9.97a	4.68b	**	ns	ns	ns	ns
Furfural	0.44	0.49	ns	0.44	0.50	ns	1.08a	0.21b	0.12b	***	ns	ns	ns	ns

(Continues on the next page).

Table S1. (Continued).

Compound	Treatment (T)			Site (S)			Year (Y)				Y × T	S × T	Y × S	Y × S × T
	UNT	CT	Sig. F [1]	FG	FCO	Sig. F	2017	2018	2019	Sig. F				
Alcohols	2388.08b	2591.65a	*	2335.76b	2643.97a	***	1829.44b	1382.90c	4257.26a	***	ns	ns	***	ns
n-Hexanol	81.63	83.19	ns	63.33b	101.49a	***	157.24a	41.29b	48.70b	***	ns	ns	***	ns
trans-3-Hexenol	1.55	1.61	ns	1.14b	2.02a	***	2.59a	1.06b	1.09b	***	ns	ns	***	ns
1-Octanol	10.27	9.46	ns	9.65	10.08	ns	9.25	11.39	8.96	ns	ns	ns	ns	ns
Isobutanol	102.81	98.72	ns	90.30b	111.22a	*	85.49b	118.73a	98.06b	**	ns	ns	*	*
Methionol	4.78	5.12	ns	4.36b	5.54a	***	6.62a	4.10b	4.13b	***	ns	ns	***	ns
Isoamyl alcohol	1462.30b	1595.95a	**	1431.83b	1626.42a	**	658.32b	645.54b	3283.53a	***	**	ns	**	ns
3-Methyl-1-pentanol	7.10	8.36	ns	6.50b	8.96a	***	7.15	8.06	7.99	ns	ns	ns	***	ns
2,3-Butanediol (isomer 1)	16.85	17.59	ns	18.64	15.80	ns	21.42a	13.12b	17.13b	**	ns	ns	ns	ns
2,3-Butanediol (isomer 2)	4.35	5.15	ns	5.26	4.24	ns	5.71	4.45	4.09	ns	ns	ns	ns	ns
cis-3-Hexenol	696.46b	766.49a	*	704.75	758.20	ns	875.65a	535.17c	783.60b	***	ns	ns	***	ns
Esters	2286.64	2414.24	ns	2363.73	2337.15	ns	4111.28a	1809.03b	1131.00c	***	ns	ns	ns	ns
Ethyl acetate	93.05b	112.05a	ns	86.36b	118.74a	**	127.85a	67.37b	112.44a	***	ns	ns	ns	ns
Ethyl butyrate	27.66	30.36	ns	28.01	30.01	ns	32.00	26.89	28.13	ns	ns	ns	ns	ns
Isopentyl acetate	208.71	226.90	ns	212.83	222.78	ns	310.17a	151.91b	191.33b	***	ns	ns	ns	ns
Hexyl acetate	50.03	47.89	ns	41.03b	56.89a	*	114.19a	13.12b	19.58b	***	ns	ns	***	ns
Methyl caproate	0.44	0.41	ns	0.45	0.40	ns	0.36	0.43	0.49	ns	ns	ns	ns	ns
Ethyl hexanoate	705.59	733.38	ns	713.18	725.79	ns	974.87a	726.63b	456.96c	***	ns	ns	ns	ns
Ethyl lactate	3.24	3.50	ns	3.33	3.40	ns	4.70a	2.49b	2.92b	***	ns	ns	***	ns
Methyl octanoate	1.26	1.29	ns	1.21	1.34	ns	2.15a	1.04b	0.64b	***	ns	ns	ns	ns
Ethyl octanoate	687.39	743.26	ns	679.43	751.23	ns	1641.87a	306.94b	197.17b	***	ns	ns	ns	ns
Isoamyl lactate	0.65	0.73	ns	0.56b	0.82a	***	0.52b	0.83a	0.72a	***	ns	ns	***	ns
Methyl decanoate	0.12	0.12	ns	0.11	0.13	ns	0.26a	0.04b	0.06b	***	ns	ns	ns	ns

(Continues on the next page).

Table S1. (Continued).

Compound	Treatment (T)			Site (S)			Year (Y)				Y × T	S × T	Y × S	Y × S × T
	UNT	CT	Sig. F [1]	FG	FCO	Sig. F	2017	2018	2019	Sig. F				
Ethyl decanoate	180.36	179.37	ns	184.99	174.73	ns	387.03a	99.53b	53.03b	***	ns	ns	ns	ns
Isoamyl octanoate	3.23	3.65	ns	2.77b	4.10a	*	5.85a	3.21b	1.25c	***	ns	ns	ns	ns
Methyl ethyl succinate	128.68	118.34	ns	153.74a	93.28b	**	102.55b	267.94a	0.04c	***	ns	ns	**	ns
Diethyl succinate	60.91	64.43	ns	70.35a	54.99b	*	156.40a	28.99b	2.63c	***	ns	ns	ns	ns
Ethyl 9-decanoate	56.68	61.14	ns	101.15a	16.67b	***	104.12a	58.89b	13.72c	***	ns	ns	**	ns
Ethyl-2-OH-4-methylpentanoate	1.39	1.28	ns	1.20b	1.48a	**	1.77a	1.48b	0.75c	***	ns	ns	***	ns
2-Phenylethyl acetate	58.91	61.97	ns	64.23	56.65	ns	108.44a	38.28b	34.60b	***	ns	ns	ns	ns
Ethyl hydrogen succinate	3.94	4.40	ns	3.53b	4.80a	*	5.87a	6.18a	0.44b	***	ns	ns	***	ns
Methyl salicylate	9.21	5.66	ns	9.00	5.87	ns	19.98a	1.88b	0.46b	**	ns	ns	ns	ns
Ethyl hydroxybutanoate	5.20	6.26	ns	6.28	5.18	ns	10.34a	4.98b	1.87c	***	ns	ns	ns	ns
Acids	1702.88	1551.69	ns	1489.00	1765.56	ns	2533.89a	732.68 c	1615.27b	***	ns	ns	ns	ns
Acetic acid	36.27	36.28	ns	32.45	40.10	ns	16.89b	46.92a	45.02a	***	ns	ns	*	*
Butyric acid	5.38	5.56	ns	5.06	5.88	ns	5.28	6.28	4.85	ns	ns	ns	ns	*
3-Methylbutyric acid	18.15	19.16	ns	17.03b	20.27a	*	23.79a	19.80b	12.37c	***	ns	ns	*	ns
Hexanoic acid	106.63	105.41	ns	102.06	109.98	ns	154.54a	65.16c	98.36b	***	ns	ns	*	ns
Octanoic acid	462.97	453.73	ns	436.30	480.41	ns	729.19a	230.62c	415.25b	***	ns	ns	ns	ns
Nonanoic acid	32.07	25.98	ns	28.99	29.07	ns	6.24b	6.34b	74.51a	***	ns	ns	ns	ns
Decanoic acid	1016.65	882.67	ns	842.94	1056.38	ns	1557.00a	346.49c	945.50b	***	ns	ns	ns	ns
Benzoic acid	6.75	5.99	ns	5.02	7.72	ns	15.44a	2.62b	1.06b	*	ns	ns	ns	ns
Dodecanoic acid	18.00	16.90	ns	19.15	15.75	ns	25.53a	8.46b	18.36ab	*	ns	ns	*	*
Ketones	17.75	19.38	ns	18.24	18.89	ns	20.08	15.45	20.16	ns	ns	ns	ns	ns
2-Methylthiolan-3-one	2.23	2.42	ns	2.81a	1.84b	*	1.99	2.79	2.20	ns	ns	ns	ns	ns
Isophorone	15.52	16.96	ns	15.43	17.05	ns	18.1	12.67	17.96	ns	ns	ns	ns	ns

(Table footer continues the next page).

[1] Data were analyzed by three-ways ANOVA (ns, not significant; *, $p < 0.05$; **, $p < 0.01$; ***, $p < 0.001$), and when differences were significant, the means were separated using Student Newman Keuls test ($p < 0.05$). [2] Different letters (a, b, c) identify significantly different means. UNT, untreated control; CT, cluster thinning; FG, Friuli Grave; FCO, Friuli Colli Orientali. All the concentrations are expressed in μg/L as IS 2-octanol.

Table S2. Impact of cluster thinning treatment, vineyard site and harvest season on the volatile profile of Ribolla Gialla sparkling wines.

Compound	Treatment (T)			Site (S)			Year (Y)				Y × T	S × T	Y × S	Y × S × T
	UNT	CT	Sig. F [1]	FG	FCO	Sig. F	2017	2018	2019	Sig. F				
Monoterpenes	24.54	25.54	ns	25.57	24.51	ns	21.44b	22.21b	31.48a	***	ns	***	ns	ns
β-Myrcene	0.70b [2]	0.80a	**	0.74	0.75	ns	0.70b	0.70b	0.83a	**	ns	ns	ns	ns
Limonene	2.32	2.45	ns	2.50	2.27	ns	2.36	2.40	2.41	ns	ns	ns	ns	ns
Linalool	4.55b	4.95a	*	4.59b	4.90a	*	4.49b	5.11a	4.64b	ns	ns	*	ns	ns
Geraniol	7.85	7.27	ns	7.33	7.80	ns	8.18	7.13	7.38	ns	ns	*	ns	ns
Citronellol	1.41b	1.60a	*	1.37b	1.64a	**	1.44b	1.19c	1.88a	***	ns	**	ns	**
Nerol	3.04	2.96	ns	2.80b	3.20a	***	2.34c	2.66b	4.00a	***	ns	***	*	**
Terpinen-4-ol	0.54	0.55	ns	0.52	0.57	ns	0.45b	0.61a	0.57a	**	ns	ns	ns	ns
α-Terpineol	4.13	4.97	ns	5.73a	3.38b	***	1.47b	2.42b	9.77a	***	ns	***	ns	ns
Norisoprenoids	16.17	16.63	ns	15.16b	17.63a	***	18.00a	11.92b	19.28a	***	ns	***	ns	*
Vitispirane	0.62	0.79	ns	0.76	0.66	ns	0.81	0.60	0.72	ns	ns	ns	ns	ns
TDN	0.99	1.08	ns	0.92b	1.15a	**	0.95b	0.40c	1.75a	***	ns	ns	ns	ns
β-Damascenone	13.62	13.88	ns	12.57b	14.93a	***	15.21a	10.00b	16.05a	***	ns	***	**	**
Actinidiol (isomer 1)	0.40	0.37	ns	0.39	0.39	ns	0.44a	0.40b	0.32c	***	ns	***	ns	ns
Actinidiol (isomer 2)	0.53	0.50	ns	0.52	0.51	ns	0.59a	0.53b	0.43c	***	ns	***	ns	ns
Aldehydes	417.83b	474.89a	*	423.12	469.60	ns	499.58a	360.28b	479.23a	***	ns	ns	ns	ns
Hexanal	0.09	0.07	ns	0.08	0.09	ns	0.12a	0.05c	0.08b	***	ns	ns	ns	ns
trans-2-Hexenal	388.74b	441.41a	*	394.47	435.68	ns	464.22a	334.23b	446.77a	***	ns	ns	ns	ns
Nonanal	3.24	2.92	ns	3.01	3.15	ns	2.86b	2.49b	3.89a	*	ns	*	ns	ns
Benzaldehyde	6.69	7.00	ns	6.42	7.27	ns	8.75a	5.28c	6.51b	***	ns	ns	ns	ns
3,4-Dimethyl benzaldehyde	1.43	1.52	ns	1.43	1.52	ns	1.69a	1.00b	1.73a	***	ns	ns	ns	ns
Acetaldehyde	16.18b	20.48a	**	16.14b	20.52a	**	20.41	16.14	18.44	ns	ns	ns	ns	ns
Furfural	1.47	1.49	ns	1.59	1.38	ns	1.53a	1.09b	1.82a	***	ns	ns	ns	**

(Continues on the next page)

Table S2. (Continued).

Compound	Treatment (T)			Site (S)			Year (Y)				Y × T	S × T	Y × S	Y × S × T
	UNT	CT	Sig. F [1]	FG	FCO	Sig. F	2017	2018	2019	Sig. F				
Alcohols	3678.56b	4086.47a	**	3791.62	3973.41	ns	3975.01a	3538.87b	4133.67a	**	ns	ns	ns	ns
n-Hexanol	75.74	79.19	ns	61.63b	93.30a	***	144.07a	30.64c	57.68b	***	ns	***	ns	ns
trans-3-Hexenol	1.76	1.88	ns	1.36b	2.28a	***	3.04a	1.02c	1.40b	***	ns	***	ns	ns
1-octanol	11.20	10.44	ns	10.74	10.90	ns	10.99	10.39	11.09	ns	**	**	ns	ns
Iso-butanol	106.05b	119.85a	*	109.58	116.32	ns	110.03b	95.94b	132.87a	***	ns	ns	ns	**
Methionol	2.31	2.85	ns	2.76	2.40	ns	2.24	2.84	2.66	ns	*	ns	ns	ns
Isoamyl alcohol	2749.83b	3073.61a	**	2845.52	2977.92	ns	2893.35b	2669.83b	3171.99a	**	ns	ns	ns	ns
3-Methyl-1-pentanol	9.13b	10.86a	**	10.03	9.96	ns	8.90b	10.59a	10.50a	*	ns	ns	ns	ns
2,3-Butanediol (isomer 1)	5.55a	2.44b	*	3.32	4.66	ns	10.39a	0.59b	1.00b	***	ns	ns	*	ns
2,3-Butanediol (isomer 2)	2.22a	1.23b	*	1.58	1.87	ns	3.87a	0.46b	0.85b	***	ns	ns	*	ns
cis-3-Hexenol	0.29b	0.51a	*	0.47	0.32	ns	0.45	0.38	0.36	ns	ns	ns	ns	ns
trans-2-Hexenol	1.04	1.49	ns	1.05	1.48	ns	1.39	1.13	1.27	ns	ns	ns	ns	ns
2-Phenylethanol	714.77b	784.12a	**	745.10	753.79	ns	788.14	716.56	743.64	ns	ns	ns	ns	ns
Esters	2667.36	2978.17	ns	2912.35	2733.18	ns	2484.72b	2539.83b	3443.76a	***	ns	ns	ns	ns
Ethyl acetate	299.39	347.16	ns	298.65	347.9	ns	307.33b	237.68c	424.81a	***	ns	ns	ns	ns
Ethyl butyrate	42.62	46.24	ns	46.86	42.00	ns	36.59b	41.44b	55.26a	***	ns	ns	ns	ns
Isopentyl acetate	189.33	193.35	ns	205.63	177.05	ns	138.94b	189.43c	245.65a	***	ns	ns	ns	ns
Hexyl acetate	22.09	21.81	ns	25.96a	17.93b	*	20.84ab	16.46b	28.53a	*	ns	***	ns	ns
Methyl caproate	0.58	0.63	ns	0.60	0.61	ns	0.48b	0.57b	0.76a	***	ns	ns	ns	ns
Ethyl hexanoate	947.62	1044.37	ns	1020.51	971.48	ns	1000.85	940.15	1046.99	ns	ns	ns	ns	ns
Ethyl lactate	15.40b	17.84a	**	17.04	16.20	ns	18.74a	14.52c	16.60b	***	ns	**	ns	ns
Methyl octanoate	3.06	3.17	ns	3.18	3.05	ns	2.41b	3.26a	3.68a	***	ns	ns	ns	ns

(Continues on the next page

Table S2. (Continued).

Compound	Treatment (T)			Site (S)			Year (Y)				Y×T	S×T	Y×S	Y×S×T
	UNT	CT	Sig. F [1]	FG	FCO	Sig. F	2017	2018	2019	Sig. F				
Ethyl octanoate	587.02	659.98	ns	627.28	619.72	ns	462.69c	627.70b	780.11a	***	ns	ns	ns	ns
Isoamyl lactate	2.31	2.40	ns	2.31	2.40	ns	2.46a	2.12b	2.49a	**	ns	*	ns	ns
Methyl decanoate	0.28	0.31	ns	0.28	0.30	ns	0.23b	0.22b	0.43a	***	ns	ns	ns	ns
Ethyl decanoate	168.27	188.86	ns	183.12	174.01	ns	27.45c	168.00b	340.25a	***	ns	ns	ns	ns
Isoamyl octanoate	2.47	2.59	ns	2.55	2.51	ns	1.91b	2.54a	3.14a	**	ns	ns	ns	ns
Methyl ethyl succinate	106.51	121.36	ns	117.71	110.17	ns	109.76b	81.40c	150.65a	***	ns	ns	ns	ns
Diethyl succinate	89.89b	102.10a	**	96.81	95.18	ns	144.32a	98.95b	44.71c	***	ns	ns	ns	ns
Methyl decanoate	0.28	0.31	ns	0.28	0.30	ns	0.23b	0.22b	0.43a	***	ns	ns	ns	ns
Ethyl decanoate	168.27	188.86	ns	183.12	174.01	ns	27.45c	168.00b	340.25a	***	ns	ns	ns	ns
Isoamyl octanoate	2.47	2.59	ns	2.55	2.51	ns	1.91b	2.54a	3.14a	**	ns	ns	ns	ns
Methyl ethyl succinate	106.51	121.36	ns	117.71	110.17	ns	109.76b	81.40c	150.65a	***	ns	ns	ns	ns
Diethyl succinate	89.89b	102.10a	**	96.81	95.18	ns	144.32a	98.95b	44.71c	***	ns	ns	ns	ns
Ethyl 9-decanoate	118.88b	148.53a	**	185.20a	82.21b	***	151.24b	15.40c	234.47a	***	ns	***	ns	ns
Ethyl-2-OH-4-methylpentanoate	3.21	2.94	ns	3.05	3.10	ns	2.62b	3.68a	2.92b	***	ns	ns	ns	ns
2-Phenylethyl acetate	43.50	47.62	ns	48.41	42.71	ns	37.47b	51.25a	47.96a	**	ns	ns	ns	ns
Ethyl hydrogen succinate	16.76	19.68	ns	19.74	16.7	ns	12.69b	35.27a	6.69b	***	ns	ns	ns	ns
Methyl salicylate	5.38	3.91	ns	4.65	4.64	ns	3.58b	7.58a	2.79b	**	ns	ns	ns	ns
Ethyl hydroxybutanoate	2.01b	2.51a	*	2.06	2.46	ns	1.14b	1.62b	4.02a	***	ns	ns	ns	*
Ethyl dodecanoate	0.78	0.83	ns	0.76	0.85	ns	0.97a	0.58b	0.86a	***	ns	*	ns	ns
Isobutyl acetate	1.02	1.69	ns	1.27	1.43	ns	1.56	1.15	1.34	ns	*	ns	ns	ns
Acids	2280.49	2267.72	ns	2296.39	2251.83	ns	1840.70b	2718.65a	2262.97ab	**	ns	ns	ns	ns
Acetic acid	33.76b	41.79a	*	33.27b	42.28a	**	21.45c	36.02b	55.85a	***	ns	*	*	ns
Butyric acid	7.30	7.17	ns	7.45	7.01	ns	6.08c	7.34b	8.29a	***	ns	ns	ns	ns

(Continues on the next page)

Table S2. (Continued)

Compound	Treatment (T)			Site (S)			Year (Y)				Y×T	S×T	Y×S	Y×S×T
	UNT	CT	Sig. F [1]	FG	FCO	Sig. F	2017	2018	2019	Sig. F				
Butyric acid	7.30	7.17	ns	7.45	7.01	ns	6.08c	7.34b	8.29a	***	ns	ns	ns	ns
Hexanoic acid	120.51	124.46	ns	121.49	123.47	ns	124.04	115.83	127.59	ns	ns	ns	ns	ns
Octanoic acid	455.15	466.16	ns	460.77	460.55	ns	388.98b	443.06b	549.94a	***	ns	ns	ns	ns
Nonanoic acid	49.37	43.05	ns	45.50	46.92	ns	61.10a	20.42b	57.11a	**	*	ns	ns	*
Decanoic acid	1548.92	1517.93	ns	1558.47	1508.38	ns	1185.85b	2005.13a	1409.30b	**	ns	ns	ns	ns
Benzoic acid	2.63	3.22	ns	3.00a	2.85b	*	2.84	2.99	2.94	ns	ns	ns	ns	ns
Dodecanoic acid	43.63	44.51	ns	47.19	40.95	ns	30.19b	68.46a	33.55b	***	ns	ns	ns	ns
Ketones	139.98	147.75	ns	145.05	142.68	ns	159.06a	134.54b	137.98b	**	ns	ns	ns	ns
2-Methylthiolan-3-one	2.30	2.71	ns	3.21a	1.80b	***	0.83c	3.79a	2.89b	***	ns	***	ns	ns
Isophorone	137.68	145.03	ns	141.84	140.88	ns	158.24a	130.75b	135.09b	**	ns	ns	ns	ns

[1] Data were analyzed by three-ways ANOVA (ns, not significant; *, $p < 0.05$; **, $p < 0.01$; ***, $p < 0.001$), and when differences were significant, the means were separated using Student Newman Keuls test ($p < 0.05$). [2] Different letters (a, b, c) identify significantly different means. UNT, untreated control; CT, cluster thinning; FG, Friuli Grave; FCO, Friuli Colli Orientali. All the concentrations are expressed in µg/L as IS 2-octanol, except *cis*-3-hexenol, *trans*-2-hexenol, and isobutyl acetate that are reported in ng/L.

Table S3. Impact of cluster thinning treatment, vineyard site and harvest season on the lipid profile of Ribolla Gialla base wines.

Compound	Treatment (T)			Site (S)			Year (Y)				Y×T	S×T	Y×S	Y×S×T
	UNT	CT	Sig. F [1]	FG	FCO	Sig. F	2017	2018	2019	Sig. F				
Glycerolipids	0.26	0.26	ns	0.25	0.26	ns	0.27	0.24	0.26	ns	ns	ns	ns	ns
1-Linoleoyl-rac-glycerol	0.23	0.23	ns	0.22	0.23	ns	0.23	0.21	0.24	ns	ns	ns	ns	ns
1-Oleoyl-rac-glycerol	0.02	0.02	ns	0.02	0.02	ns	0.02a [2]	0.02b	0.02a	**	ns	ns	ns	ns
1-Monopalmitoleoyl-rac-glycerol	0.01	0.01	ns	0.01	0.01	ns	0.01	0.01	0.01	ns	ns	ns	ns	ns
Sterols	0.30	0.30	ns	0.29	0.31	ns	0.32a	0.27b	0.31a	*	ns	ns	ns	ns
Ergosterol	0.28	0.27	ns	0.27	0.28	ns	0.29a	0.25b	0.29a	*	ns	ns	ns	ns
Desmosterol	0.03	0.03	ns	0.03	0.03	ns	0.03a	0.02b	0.03a	*	ns	ns	ns	ns
Fatty acids UFA	4.66	4.71	ns	4.67	4.71	ns	6.26a	3.83b	3.98b	***	ns	ns	ns	ns
Linoleic acid	0.28	0.27	ns	0.27	0.28	ns	0.36a	0.23b	0.24b	***	ns	ns	ns	ns
Linolenic acid	0.03	0.03	ns	0.03a	0.03b	*	0.03a	0.03b	0.03ab	**	ns	ns	ns	ns
Palmitoleic acid	0.14	0.16	ns	0.15	0.16	ns	0.23a	0.11b	0.11b	***	ns	ns	ns	ns
Oleic acid + *cis*-Vaccenic acid	4.21	4.25	ns	4.22	4.24	ns	5.64a	3.46b	3.59b	***	ns	ns	ns	ns
Fatty acids SFA	154.72	152.89	ns	152.80	154.81	ns	179.54a	142.44b	139.44b	***	ns	ns	ns	ns
Behenic acid	0.65	0.64	ns	0.64	0.64	ns	0.73a	0.60b	0.60b	***	ns	ns	*	ns
Stearic acid	45.70	45.97	ns	45.38	46.30	ns	54.75a	42.66b	40.10b	***	ns	ns	ns	ns
Lignoceric acid	0.34	0.34	ns	0.34	0.34	ns	0.37a	0.34ab	0.31b	*	ns	ns	ns	ns
Arachidic acid	2.24	2.26	ns	2.22	2.28	ns	2.76a	1.95b	2.03b	***	ns	ns	ns	ns
Myristic acid	1.55	1.60	ns	1.49b	1.66a	**	1.60b	1.33c	1.79a	***	ns	ns	*	ns
Palmitic acid	103.31	101.18	ns	101.84	102.66	ns	118.31a	94.73b	93.70b	***	ns	ns	ns	ns
Miristoleic acid	0.47	0.47	ns	0.46	0.48	ns	0.49a	0.42b	0.49a	*	ns	ns	ns	ns
Margaric acid	0.45	0.44	ns	0.44	0.45	ns	0.53a	0.40b	0.41b	***	ns	ns	ns	ns
Prenols	0.15	0.09	ns	0.09	0.14	ns	0.11	0.13	0.11	ns	ns	ns	ns	ns
Lupeol	0.15	0.09	ns	0.09	0.14	ns	0.11	0.13	0.11	ns	ns	ns	ns	ns

[1] Data were analyzed by three-ways ANOVA (ns, not significant; *, $p < 0.05$; **, $p < 0.01$; ***, $p < 0.001$), and when differences were significant, the means were separated using Student Newman Keuls test ($p < 0.05$). [2] Different letters (a, b, c) identify significantly different means. UNT, untreated control; CT, cluster thinning; FG, Friuli Grave; FCO, Friuli Colli Orientali. All the concentrations are expressed in mg/L.

Table S4. Impact of cluster thinning, vineyard site and harvest season on the lipid profile of Ribolla Gialla sparkling wines.

Compound	Treatment (T)			Site (S)			Year (Y)				Y×T	S×T	Y×S	Y×S×T
	UNT	CT	Sig. F [1]	FG	FCO	Sig. F	2017	2018	2019	Sig. F				
Glycerolipids	0.27	0.27	ns	0.27	0.27	ns	0.23b [2]	0.24b	0.33a	***	ns	*	ns	ns
1-Linoleoyl-*rac*-glycerol	0.23	0.23	ns	0.23	0.23	ns	0.20b	0.21b	0.28a	***	ns	ns	ns	ns
1-Oleoyl-*rac*-glycerol	0.03	0.03	ns	0.03	0.03	ns	0.03ab	0.02b	0.04a	*	ns	ns	ns	ns
1-Monopalmitoleoyl-*rac*-glycerol	7.23	8.47	ns	6.35	9.35	ns	4.76b	5.62b	13.16a	*	ns	ns	ns	ns
Sterols	0.33	0.33	ns	0.34	0.32	ns	0.29b	0.27c	0.44a	***	*	*	ns	ns
Ergosterol	0.30	0.30	ns	0.31a	0.29b	*	0.26b	0.24c	0.40a	***	ns	*	ns	ns
Desmosterol	0.03	0.03	ns	0.03	0.03	ns	0.03b	0.02b	0.04a	***	ns	ns	ns	ns
Fatty acids UFA	6.02	5.81	ns	5.84	5.99	ns	6.07a	5.45b	6.22a	***	ns	*	ns	ns
Linoleic acid	0.32	0.33	ns	0.33	0.33	ns	0.29b	0.31b	0.37a	***	ns	ns	ns	ns
Linolenic acid	0.03	0.03	ns	0.03	0.03	ns	0.03b	0.03b	0.04a	***	ns	ns	ns	ns
Palmitoleic acid	0.41a	0.31b	**	0.33b	0.40a	*	0.55a	0.33b	0.21c	***	ns	ns	*	*
Oleic acid + *cis*-Vaccenic acid	5.25	5.14	ns	5.16	5.23	ns	5.20b	4.79c	5.60a	***	ns	*	**	ns
Fatty acids SFA	205.53	198.55	ns	203.76	200.32	ns	202.31b	184.40c	219.42a	***	ns	ns	ns	ns
Behenic acid	0.81	0.81	ns	0.81	0.80	ns	0.76b	0.71b	0.96a	***	ns	ns	ns	ns
Stearic acid	62.61	60.38	ns	61.88	61.11	ns	60.79b	56.35c	67.35a	***	ns	ns	ns	ns
Lignoceric acid	0.46	0.44	ns	0.45	0.45	ns	0.49a	0.42b	0.45b	***	ns	ns	ns	ns
Arachidic acid	2.81	2.80	ns	2.85	2.77	ns	2.68b	2.50c	3.24a	***	ns	ns	ns	ns
Myristic acid	1.84	1.81	ns	1.81	1.84	ns	1.76b	1.68b	2.04a	***	ns	ns	ns	ns
Palmitic acid	135.95	131.28	ns	134.92	132.31	ns	134.88b	121.84c	144.12a	***	ns	ns	ns	ns
Miristoleic acid	0.49	0.50	ns	0.51	0.49	ns	0.41b	0.41b	0.68a	***	ns	ns	ns	ns
Margaric acid	0.56	0.53	ns	0.54	0.56	ns	0.55b	0.50c	0.60a	***	ns	ns	ns	ns
Fatty esters	16.29	9.70	ns	9.45	16.54	ns	24.22a	9.98b	4.79c	*	ns	ns	ns	ns
Ethyl stearate	16.29	9.70	ns	9.45	16.54	ns	24.22a	9.98b	4.79c	*	ns	ns	ns	ns

(Continues on the next page)

Table S4. (Continued).

Compound	Treatment (T)			Site (S)			Year (Y)				Y×T	S×T	Y×S	Y×S×T
	UNT	CT	Sig. F [1]	FG	FCO	Sig. F	2017	2018	2019	Sig. F				
Prenols	0.14	0.11	ns	0.14	0.11	ns	0.11ab	0.07b	0.18a	*	ns	ns	ns	ns
Lupeol	0.14	0.11	ns	0.14	0.11	ns	0.11ab	0.07b	0.18a	*	ns	ns	ns	ns

[1] **Data were analyzed by three-ways ANOVA (ns, not significant; *, $p < 0.0$**5; **, $p < 0.01$; ***, $p < 0.001$), and when differences were significant, the means were separated using Student Newman Keuls test ($p < 0.05$). [2] Different letters (a, b, c) identify significantly different means. UNT, untreated control; CT, cluster thinning; FG, Friuli Grave; FCO, Friuli Colli Orientali. All the concentrations are expressed in mg/L except 1-monopalmitoleoyl-rac-glycerol, fatty esters, and ethyl stearate that are reported in μg/L.

Table S5. Impact of cluster thinning, vineyard site and harvest season on the aromatic amino acid metabolites profile of Ribolla Gialla base wines.

Compound[3]	Treatment (T)			Site (S)			Year (Y)				Y×S	Y×T	S×T	Y×S×T
	UNT	CT	Sig. F [1]	FG	FCO	Sig. F	2017	2018	2019	Sig. F				
TYR	3.06	3.15	ns	2.51b [2]	3.70a	**	4.15a	2.36b	2.89b	***	***	ns	ns	ns
PHE	0.99	0.89	ns	0.46b	1.42a	***	2.00a	0.35b	0.45b	***	***	ns	ns	ns
TRP	0.26	0.30	ns	0.27	0.29	ns	0.20	0.36	0.29	ns	ns	ns	*	*
KYNA	6.09	8.24	ns	4.96b	9.38a	**	9.66a	1.50b	10.79a	***	*	ns	ns	ns
NIC	0.39	0.25	ns	0.32	0.32	ns	0.28	0.26	0.41	ns	*	ns	ns	ns
TRP-EE	0.27	0.33	ns	0.30	0.30	ns	0.23b	0.39a	0.27b	***	*	*	ns	***
TYR-EE	6.50	5.50	ns	6.62	5.38	ns	10.91a	2.85b	4.51b	***	ns	ns	ns	ns
N-TYR-EE	0.28b	0.43a	**	0.36	0.34	ns	0.41a	0.26b	0.39a	*	**	ns	ns	ns
TYL	14.08	14.08	ns	15.26a	12.90b	**	15.26	13.10	14.02	ns	**	ns	ns	ns
OH-TYL	0.34	0.38	ns	0.38	0.34	ns	0.36b	0.27c	0.45a	***	**	ns	ns	***
Ph-AA	0.28	0.24	ns	0.26	0.26	ns	0.28	0.20	0.29	ns	ns	ns	ns	ns
TOL	0.35	0.39	ns	0.40	0.33	ns	0.27b	0.39a	0.46a	**	**	ns	ns	ns
IAA	0.49	0.41	ns	0.36b	0.54a	*	0.60a	0.34b	0.43b	*	ns	ns	ns	ns
ILA	0.39	0.34	ns	0.40	0.32	ns	0.47	0.30	0.30	ns	ns	ns	ns	ns
ILA-GLU[4]	0.99b	1.21a	**	1.21a	0.99b	**	0.74c	1.14b	1.38a	***	***	ns	**	***
N-SER	0.50a	0.42b	**	0.48	0.44	ns	0.42	0.48	0.46	ns	ns	ns	ns	*
Ph-LA	0.64	0.69	ns	0.72a	0.62b	**	0.54b	0.71a	0.76a	***	***	ns	*	ns
TOL-SO_3H	0.42	0.37	ns	0.42	0.38	ns	0.72a	0.27b	0.20b	***	ns	ns	ns	ns
ABA	0.39	0.42	ns	0.44a	0.37b	*	0.56a	0.31b	0.34b	***	***	ns	ns	ns
ABA-GLU	0.40	0.39	ns	0.33	0.46	ns	0.41	0.34	0.43	ns	*	ns	*	ns

[1] Data were analyzed by three-ways ANOVA (ns, not significant; *, $p < 0.05$; **, $p < 0.01$; ***, $p < 0.001$), and when differences were significant, the means were separated using Student Newman Keuls test ($p < 0.05$). [2] Different letters (a, b, c) identify significantly different means. UNT, untreated control; CT, cluster thinning; FG, Friuli Grave; FCO, Friuli Colli Orientali. [3] (TYR) Tyrosine; (PHE) Phenylalanine; (TRP) Tryptophan; (KYNA) Kynurenic acid; (NIC) Nicotinamide; (TRP-EE) Tryptophan ethyl ester; (TYR-EE) Tyrosine ethyl ester; (N-TYR-EE) N-acetyl tyrosine ethyl ester; (TYL) Tyrosol; (OH-TYL) Hydroxytyrosol; (Ph-AA) Phenyl acetic acid; (TOL) Tryptophol; (IAA) Indole 3-acetic acid; (ILA) Indole 3-lactic acid; (ILA-GLU) Indole 3-lactic acid glucoside; (N-SER) N-acetyl serotonin; (Ph-LA) Phenyl lactic acid; (TOL-SO3H) Tryptophol-2-sulfonic acid; (ABA) Abscisic acid; (ABA-GLU) Abscisic acid glucoside. [4] Quantified as ILA. All the concentrations are expressed in mg/L.

Table S6. Impact of cluster thinning, vineyard site and harvest season on the aromatic amino acid metabolites profile of Ribolla Gialla sparkling wines.

Compound[3]	Treatment (T)			Site (S)			Year (Y)				Y×S	Y×T	S×T	Y×S×T
	UNT	CT	Sig. F [1]	FG	FCO	Sig. F	2017	2018	2019	Sig. F				
TYR	3.62b [2]	5.26a	**	3.80b	5.07a	*	4.50	4.59	4.23	ns	*	*	**	***
PHE	2.68	2.36	ns	1.48b	3.56a	***	4.32a	2.43b	0.81c	***	***	ns	ns	*
TRP	0.34	0.28	ns	0.31	0.31	ns	0.35a	0.20b	0.38a	**	*	*	*	ns
KYNA	7.80	8.50	ns	5.32b	10.98a	**	12.32a	2.34b	9.78a	***	*	ns	ns	ns
NIC	0.48	0.39	ns	0.45	0.42	ns	0.46a	0.21b	0.63a	***	*	ns	ns	ns
TRP-EE	0.40a	0.21b	*	0.28	0.33	ns	0.24	0.40	0.29	ns	ns	ns	ns	ns
TYR-EE	14.99	12.41	ns	9.94b	17.47a	***	24.65a	6.49b	9.98b	***	**	ns	ns	ns
N-TYR-EE	0.29b	0.53a	*	0.39	0.43	ns	0.40	0.32	0.52	ns	ns	ns	ns	ns
TYL	15.25	15.65	ns	14.59	16.31	ns	16.78	15.80	13.77	ns	ns	ns	ns	ns
OH-TYL	0.31	0.31	ns	0.24b	0.38a	***	0.31ab	0.26b	0.35a	**	***	ns	ns	ns
Ph-AA	0.34	0.31	ns	0.34	0.31	ns	0.24	0.34	0.39	ns	ns	ns	**	ns
TOL	0.49	0.54	ns	0.51	0.51	ns	0.33c	0.73a	0.48b	***	ns	ns	ns	ns
IAA	0.43a	0.24b	*	0.33	0.33	ns	0.44	0.21	0.35	ns	ns	ns	ns	ns
ILA	0.32	0.41	ns	0.31	0.42	ns	0.40	0.36	0.33	ns	ns	ns	ns	ns
ILA-GLU[4]	1.31	1.27	ns	1.46a	1.13b	**	0.95b	1.37a	1.56a	***	ns	ns	ns	ns
N-SER	0.44	0.42	ns	0.47	0.39	ns	0.46	0.42	0.41	ns	ns	ns	ns	ns
Ph-LA	0.84	0.89	ns	0.86	0.88	ns	0.73b	1.15a	0.72b	***	**	*	ns	ns
TOL-SO_3H	0.60	0.53	ns	0.41b	0.72a	***	1.00a	0.44b	0.26b	***	ns	ns	ns	ns
2AA	0.38	0.36	ns	0.47a	0.27b	**	0.33b	0.48a	0.30b	*	*	ns	ns	ns
ABA	0.51	0.59	ns	0.58	0.53	ns	0.83a	0.43b	0.40b	***	***	ns	ns	ns
ABA-GLU	0.41	0.35	ns	0.38	0.39	ns	0.42	0.42	0.31	ns	ns	ns	ns	ns

[1] Data were analyzed by three-ways ANOVA (ns, not significant; *, $p < 0.05$; **, $p < 0.01$; ***, $p < 0.001$), and when differences were significant, the means were separated using Student Newman Keuls test ($p < 0.05$). [2] Different letters (a, b, c) identify significantly different means. UNT, untreated control; CT, cluster thinning; FG, Friuli Grave; FCO, Friuli Colli Orientali. [3] (TYR) Tyrosine; (PHE) Phenylalanine; (TRP) Tryptophan; (KYNA) Kynurenic acid; (NIC) Nicotinamide; (TRP-EE) Tryptophan ethyl ester; (TYR-EE) Tyrosine ethyl ester; (N-TYR-EE) N-acetyl tyrosine ethyl ester; (TYL) Tyrosol; (OH-TYL) Hydroxytyrosol; (Ph-AA) Phenyl acetic acid; (TOL) Tryptophol; (IAA) Indole 3-acetic acid; (ILA) Indole 3-lactic acid;

(ILA GLU) Indole 3-lactic acid glucoside; (N-SER) N-acetyl serotonin; (Ph-LA) Phenyl lactic acid; (TOL-SO3H) Tryptophol-2-sulfonic acid; (2AA) 2-Aminoacetophenone; (ABA) Abscisic acid; (ABA-GLU) Abscisic acid glucoside. [4] Quantified as ILA. All the concentrations are expressed in mg/L.

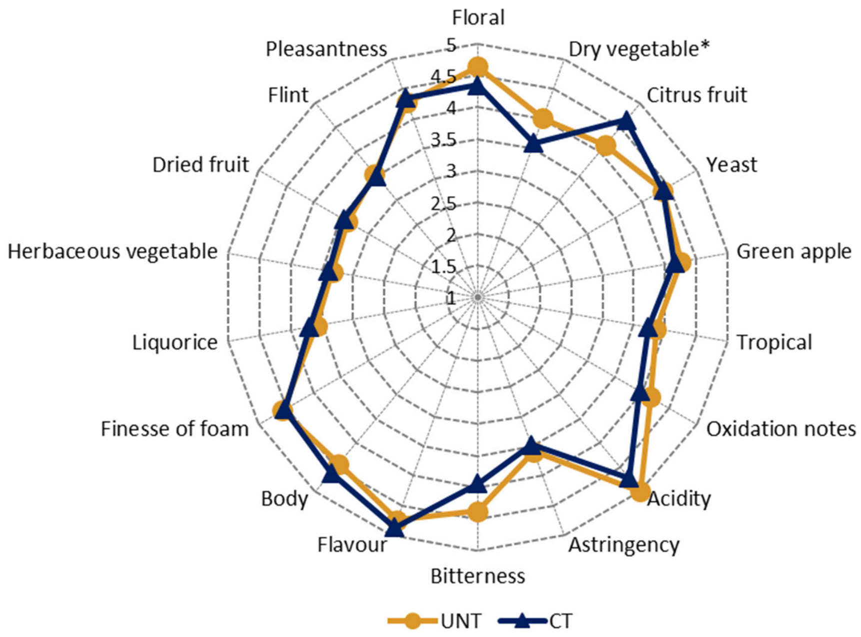

Figure S2. Effect of cluster thinning on the organoleptic characteristics of Ribolla Gialla sparkling wines. The average values were obtained from 2017–2019 and FG-FCO vineyard sites. Yellow and blue line represent untreated (UNT) and treated (CT) samples, respectively. Asterisks (*) indicate statistical significance ($p < 0.05$) for each sensory attribute.

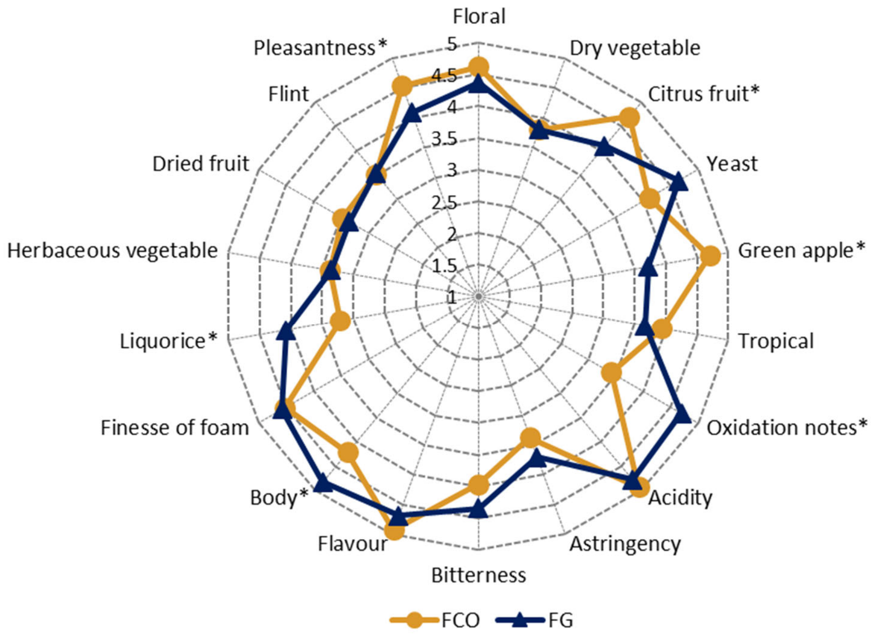

Figure S3. Effect of production site on the organoleptic characteristics of Ribolla Gialla sparkling wines. The average values were obtained from 2017–2019 of both UNT and CT samples. Yellow and blue line represent Friuli Colli Orientali (FCO) and Friuli Grave (FG) samples, respectively. Asterisks (*) indicate statistical significance ($p < 0.05$) for each sensory attribute.

Table S7. Retention indices and identification method used for VOCs analysis in base wines and sparkling wines.

Compound	RT (min)	RI_{exp}	RI_{lit}	IM	Literature
Monoterpenes					
β-Myrcene	8.59	1159	1173	MS RI STD	[62]
Limonene	9.32	1187	1204	MS RI STD	[59]
Linalool	17.60	1546	1555	MS RI STD	[60]
Geraniol	23.28	1876	1850	MS RI STD	[61]
Citronellol	21.81	1766	1777	MS RI STD	[62]
Nerol	21.73	1761	1777	MS RI STD	[63]
Terpinen-4-ol	18.69	1600	1618	MS RI STD	[64]
α-Terpineol	20.54	1696	1679	MS RI STD	[65]
Norisoprenoids					
Vitispirane	17.15	1524	1505	MS RI	[86]
TDN	21.44	1745	1732	MS RI	[60]
β-Damascenone	22.81	1833	1857	MS RI STD	[66]
Actinidiol (isomer 1)	24.65	2005	-	MS	-
Actinidiol (isomer 2)	24.88	2018	-	MS	-
Aldehydes					
Hexanal	6.55	1081	1072	MS RI STD	[67]
trans-2-Hexenal	10.73	1242	1235	MS RI STD	[68]
Nonanal	14.32	1392	1397	MS RI	[69]
Benzaldehyde	17.08	1520	1507	MS RI STD	[70]
3,4-Dimethyl benzaldehyde	22.23	1789	1790	MS RI	[71]
Acetaldehyde	1.72	549	-	MS	-
Furfural	15.86	1463	1460	MS RI	[72]
Alcohols					
n-Hexanol	13.40	1353	1358	MS RI STD	[70]
trans-3-Hexenol	13.63	1363	1374	MS RI STD	[65]
1-Octanol	17.81	1556	1562	MS RI	[70]
Iso-butanol	7.14	1104	1114	MS RI STD	[73]
Methionol	20.88	1715	1711	MS RI	[74]
Isoamyl alcohol	10.11	1218	1209	MS RI STD	[75]
3-Methyl-1-pentanol	12.76	1325	1316	MS RI STD	[74]
2,3-Butanediol (isomer 1)	17.41	1537	1529	MS RI	[76]
2,3-Butanediol (isomer 2)	18.16	1574	1583	MS RI	[75]
cis-3-Hexenol	14.57	1403	1382	MS RI STD	[76]
trans-2-Hexenol	15.06	1426	1420	MS RI STD	[73]
2-Phenylethanol	24.08	1906	1923	MS RI	[80]

(Continues on the next page).

Table S7. (Continued).

Compound	RT (min)	RI_{exp}	RI_{lit}	IM	Literature
Esters					
Ethyl acetate	2.69	892	889	MS RI	[77]
Ethyl butyrate	5.52	1040	1025	MS RI STD	[58]
Isopentyl acetate	7.60	1122	1120	MS RI STD	[59]
Hexyl acetate	11.46	1272	1295	MS RI STD	[60]
Methyl caproate	9.28	1185	1180	MS RI STD	[61]
Ethyl hexanoate	10.53	1234	1241	MS RI STD	[62]
Ethyl lactate	13.18	1343	1355	MS RI	[63]
Methyl octanoate	14.26	1390	1387	MS RI	[64]
Ethyl octanoate	15.24	1434	1453	MS RI STD	[65]
Isoamyl lactate	18.04	1568	1570	MS RI	[81]
Methyl decanoate	18.60	1596	1604	MS RI	[82]
Ethyl decanoate	19.48	1641	1643	MS RI STD	[60]
Isoamyl octanoate	19.85	1660	1654	MS RI STD	[66]
Methyl ethyl succinate	19.36	1635	1641	MS RI	[83]
Diethyl succinate	20.16	1677	1679	MS RI	[83]
Ethyl 9-decanoate	20.46	1692	1708	MS RI	[84]
Ethyl-2-OH-4-methylpentanoate	17.51	1542	1547	MS RI	[67]
2-Phenylethyl acetate	22.72	1825	1832	MS RI STD	[68]
Ethyl hydrogen succinate	31.69	-	-	MS	-
Methyl salicylate	21.99	1775	1765	MS RI STD	[70]
Ethyl hydroxybutanoate	22.51	1806	-	MS	-
Ethyl dodecanoate	22.80	1832	1850	MS RI	[85]
Isobutyl acetate	5.42	1036	1020	MS RI STD	[72]
Acids					
Acetic acid	15.66	1453	1437	MS RI STD	[70]
Butyric acid	19.26	1630	1598	MS RI STD	[72]
3-Methylbutyric acid	20.05	1671	1657	MS RI	[70]
Hexanoic acid	23.28	1876	1857	MS RI STD	[62]
Octanoic acid	26.86	-	-	MS STD	-
Nonanoic acid	28.54	-	-	MS STD	-
Decanoic acid	30.13	-	-	MS STD	-
Benzoic acid	32.47	-	-	MS	-
Dodecanoic acid	33.17	-	-	MS	-
Ketones					
2-Methylthiolan-3-one	17.16	1524	1510	MS RI	[78]
Isophorone	18.00	1566	1600	MS RI	[79]

RT, retention time in min; RI_{exp}, experimentally determined retention index; RI_{lit}, retention index reported in the literature; IM, identification method (MS, comparison of mass spectra with those reported in mass spectrum libraries; RI, comparison of order of elution with those reported in literature; STD, comparison of mass spectra and retention time with those of standard compounds).
